# Acknowledgement to Reviewers of *Cell*s in 2019

**DOI:** 10.3390/cells9010251

**Published:** 2020-01-19

**Authors:** Cells Editorial Office

**Affiliations:** MDPI AG, St. Alban-Anlage 66, 4052 Basel, Switzerland

The editorial team greatly appreciates the reviewers who have dedicated their considerable time and expertise to the journal’s rigorous editorial process over the past 12 months, regardless of whether the papers are finally published or not. In 2019, a total of 1732 papers were published in the journal, with a median time to first decision of 17.4 days and a median time from submission to publication of 37 days. The editors would like to express their sincere gratitude to the following reviewers for their generous contribution in 2019:
Aasen, TrondLisby, MichaelAbba, Mohammed L.Liss, AndrewAbbate, FrancescoListenberger, LauraAbdel Hakeem, MohamedLiu, BinAbdelalim, Essam M.Liu, ChangAbdel-Latif, AhmedLiu, Chao lienAbe, YoichiroLiu, ChunyuAberdam, DanielLiu, DelongAbouAlaiwi, WissamLiu, Guo JunAbraham, DietmarLiu, HaipengAbshagen, KerstinLiu, Jun-PingAdachi, YasushiLiu, Shing-HwaAdamec, LubomírLiu, XiangleiAdamek, AgnieszkaLiu, XiaowenAdamek, MikolajLiu, YonggangAdamowicz, JanLiu, ZhihuiAdams, BrianLivanos, PantelisAddeo, AlfredoLizano, MarcelaAdell, TeresaLizard, GérardAdhikari, NeetaLjubimov, Alexander V.Adhikary, AmitavaLlamas, BastienAdibi, JenniferLleo De Nalda, AnaAdiletta, GiuseppinaLo Sardo, FedericaAdrain, ColinLoane, DavidAfolayan, Adeleye J.Lobanova, Ekaterina S.Agarwal, Atin A.Lobo, Glenn P.Agarwal, NitinLoeffler, IvonneAgarwal, SumitLofrumento, Dario D.Aggeli, Ioanna-KaterinaLogarinho, ElsaAgresti, AlessandraLogue, SusanAguilera-Tejero, EscolasticoLoizides-Mangold, UrsulaAguirre, AitorLoman, BrettAgulló-Ortuño, M. TeresaLombardi, AngelaAhlenstiel, GoloLong, YichengAhmad, AliLonghi, Maria SerenaAhmed, FaheemuddinLongo, VitoAhmed, Shafiq U.Longone, PatriziaAhmed, ZeeshanLopalco, GiuseppeAhmetov, IldusLopes, SusanaAkamatsu, WadoLopez Perez, RamonAkbar, MohammedLopez, Jose JavierAkens, MargareteLópez, Susana B BravoAkimov, Sergey A.Lopez-Aviles, SandraAkiyoshi, BungoLópez-Camarillo, CésarAl-Ahmad, AbrahamLópez-Huertas, María RosaAlaimo, AlessandroLópez-Pedrera, CharyAlaimo, SalvatoreLoreni, FabrizioAlam, ShafiulLorente-Cebrián, SilviaAlarcon, VernadethLorenzini, AntolelloAlbano, EmanueleLorenzo González, ÓscarAlbert, Paul R.Lowe, MartinAlbertini, GiuliaLowery, JonathanAlbi, ElisabettaLowes, Lori E.Albrecht, RandyLozach, Pierre-YvesAldieri, ElisabettaLu, BingweiAldskogius, HåkanLu, Hong S.Alexander, DorotheaLu, JingAlexandrov, Ivan A.Lu, QuanlongAlexeev, AlexanderLu, ShaoyongAlfano, MassimoLu, Yong-ChenAlfarouk, Khalid OmerLucas, Andrew T.Ali, HydarLucas, RudolfAlicia, PickrellLuceri, CristinaAllard, BenoitLuconi, MichaelaAllavena, PaolaLuddi, AliceAllison, LizabethLüders, JensAllison, SimonLudidi, NdikoAllmang, ChristineLuévano-Contreras, ClaudiaAllred, Nicholette DanielleLuisetto, RobertoAlmeida, Rodrigo C.Lukas, JanAlonso, Miguel A.Lukasz, AlexanderAlpaugh, KatherineLukomski, SlawomirAltaie, AlaLuna Varo, Rosa MaríaAltier, ChristopheLund, Elizabeth K.Altmann, BrigitteLung, Shiu CheungAltmeyer, MatthiasLunov, OlegAltruda, FiorellaLuo, Jiang-YunAltucci, LuciaLuo, MaAlvarado-Kristensson, MariaLuo, XuÁlvarez Martín, JavierLuo, XuelianÁlvarez-García, ÓscarLuo, Yong-ZhangAlvarez-Lorenzo, CarmenLuo, ZonghuaAlves, C. HenriqueLupashin, VladimirAlvisi, GualtieroLusk, C. PatrickAlzaid, FawazLuth, Eric S.Åman, PierreLuwor, RodneyAmante, Edna ReginaLuxton, G.W. GantAmantini, ConsueloLy, HinhAmaral, FernandaLykke-Hartmann, KarinAmaral, RonaldoLyons, Barbara A.Amasheh, SalahM. Elkouby, YanivAmato, AntonellaM’Koma, Amosy E.Amato, RosarioMäättä, Jorma A.Ambrogini, PatriziaMaayah, Zaid H.Ambrosio, SantiagoMacaulay, ValentineAmicarelli, FernandaMacchi, BeatriceAmin, KawaMacek Jilkova, ZuzanaAmirav, IsraelMach, Robert HAmor, SandraMachaca, KhaledAmpofo, EmmanuelMachon, OndrejAmrutkar, ManojMaciejewska, DominikaAmulic, BorkoMaciukiewicz, MałgorzataAn, Hyun JooMack, Andreas F.An, YuMackay, CharlesAna, Fernández-OcañaMackenzie, GerardoAnandhan, AnnaduraiMacLean, Andrew G.Anderer, UrsulaMacNeill, AmyAndjelkovic, Anuska V.MacParland, SonyaAndo, AsakoMacpherson, James N.Andrade, JorgeMacreadie, IanAndre, HelderMacurek, LiborAndreassi, MariaMadala, Hanumantha RaoAndreoletti, PierreMadaro, LucaAndreotti, GiuseppinaMadureira, Patrícia A.Andrés-León, EduardoMaeda, AkikoAndressen, KjetilMaekawa, ShinyaAndric, SilvanaMagdinier, FrederiqueAndrysik, ZdenekMaggert, KeithAngelova, AngelinaMaggi, Leonard B.Angelucci, AdrianoMagin, ThomasAnger, MartinMagnaldo, ThierryAngius, FabrizioMagni, PaoloAnglani, FrancaMagnusson, Karl-EricAngrand, Pierre-OlivierMahadevan, DarukaAnifandis, GeorgeMahavadi, SunilaAnnamaria, FraMahevas, MatthieuAnne, MarmagneMahmood, KashifAnnesley, SarahMahmoudi, TokamehAnnicotte, Jean-SéBastienMai, SabineAnnunziata, Christina M.Maia, CláudioAnsari, ShariqMaioli, MargheritaAnsorena, EduardoMaione, Angela SerenaAntcliffe, DavidMaiti, PanchananAnticoli, SimonaMaiuri, PaoloAntognelli, CinziaMaixent, Jean MichelAntonenko, YuriMajima, Hideyuki J.Antoni, GunnarMajka, MarcinAntony, JaneMakarevich, PavelAon, Miguel A.Makareviciene, VioletaApps, RichardMakishima, MakotoAquaro, StefanoMalapelle, UmbertoAragonés, JuliánMalavolta, MarcoArai, ToshiroMaldonado, Eduardo N.Aran, GemmaMalejczyk, JacekAranda, Carlos J.Malemud, Charles J.Arango, DiegoMalerba, GiovanniAraújo, WagnerMalmström, LarsArbones-Mainar, JoseMalpeli, GiorgioArcher, FabienneMaltesen, RalucaArcucci, AlessandroMalwal, Satish R.Arechederra, MaríaMammoto, AkikoAriel, AmiramMamotte, Cyril D.Ariyoshi, WataruManavalan, BalachandranAriz, IdoiaMancinelli, RominaArmstrong, JamesMandal, AmritlalArnold, PhilippManera, ClementinaArnoult, NausicaManetti, MirkoArora, MansiManfredi, AngeloArranz, LorenaMangla, AnkitArroyo Pardo, EduardoMantadakis, ElpisArtemenko, YuliaMantamadiotis, TheoArthur, HelenMantovani, RobertoArtinger, KristinManzoor, RashidArufe, Maria C.Manzotti, GloriaAsahina, KinjiMarambaud, PhilippeAshe, AlysonMarano, GuiseppeAshrafi, HamidMarc, DanielAskjaer, PeterMarcal, HelderAslam, MuhammadMarcel, VirginieAspenström, PontusMarchetti, SandrineAsquith, Christopher R. M.Marchi, SaverioAssari, ShervinMarcuzzo, StefaniaAssunção-Miranda, IranaiaMarechal, AlexandreAstori, GiuseppeMarei, HanyAtanackovic, DjordjeMargariti, AndrianaAtawia, Reem T.Margiotta, AzzurraAtianand, ManinjayMargolis, Seth S.Atochin, DmitriyMari, MicheleAttanzio, AlessandroMariman, Edwin CmAudrito, ValentinaMarimpietri, DaniloAusserlechner, Michael J.Marín, MercedesAvezov, EdwardMarini, MarinaAvidor-Reiss, TomerMarino, FrancaAvila, MatiasMarins, MozartAvin, Keith G.Marion, EstelleAvin-Wittenberg, TamarMarkiewicz, AleksandraAvnet, SofiaMaroni, PaolaAyers, DuncanMarques, NatáliaAzad, ArunMarrazzo, PasqualeAzarin, SamiraMarsano, René MassimilianoAzimzadeh, OmidMarsh, Leigh M.Baas, PeterMarshall, Pamela A.Baba, YuhMarshall-Gradisnik, SonyaBabina, MagdaMarston, Steven B.Bacci, StefanoMartella, GiuseppinaBachschmid, MarkusMartelli, EugenioBaer, Patrick C.Martemyanov, KirillBagnoli, MarinaMartí, AmeliaBagnoli, PaolaMarti, FrancescBagyánszki, MáriaMartin, Elizabeth C.Bailer, Susanne M.Martin, FranciscoBaillie, GeorgeMartin, JacintaBajaj, JeevishaMartin, VanesaBaker, DavidMartín-Acebes, Miguel A.Balacescu, OvidiuMartín-Antonio, BeatrizBalan, SreeKumarMartinat, CécileBalasubramanian, SureshkumarMartineau, YvanBalatsos, NikolaosMartinelli, GiovanniBalcerczyk, AnetaMartínez, AlbertoBaldanzi, GianlucaMartinez, AnaBaldassarre, GustavoMartínez, ConstantinoBall, Claudia ReginaMartinez, MercedesBallini, AndreaMartínez, Víctor GBalmus, GabrielMartínez-Contreras, Rebeca D.Balogi, ZsoltMartinez-Medina, MargaritaBamford, ConnorMartínez-Sánchez, NoeliaBandapalli, Obul ReddyMartinez-Useros, JavierBandres-Ciga, SaraMartinez-Zubiaurre, ÍñigoBanerjee, DebabrataMartin-Malo, AlejandroBanerjee, KalpitaMartino, SabataBaniahmad, AriaMartinotti, SimonaBankwitz, DorotheaMartins, IanBao, Jun-XiangMartins, Mariana RenovatoBär, SéverineMaruyama, KeiBarak, BoazMarzano, CristinaBaranova, AnchaMasahiro, SatoBarbagallo, DavideMasalova, Olga V.Barbeito, Federico PMascitti, MarcoBarboni, BarbaraMasi, AnnalisaBarceló-Coblijn, GwendolynMasieri, FedericaBarile, LucioMassoumi, RaminBarnay Vedier, StephanieMastro, AndreaBaron, WiaMasui, KentaBarroso, MargaridaMasutani, MitsukoBarrow, Alexander DavidMatacchione, GiuliaBartee, EricMatallanas, DavidBartelt, AlexanderMatos, PauloBartesaghi, RenataMatouskova, PetraBartneck, MatthiasMatsuda, AkioBartoszewski, RafalMatsuda, FumioBasak, DebasishMatsugaki, AiraBashford-Rogers, RachaelMatsumura, KiyoshiBasilico, CristinaMatsuura, BunzoBasini, GiuseppinaMatsuzaki, ToshiyukiBasoli, ValentinaMatta, CsabaBasso, DanielaMattarelli, PaolaBasu, DipanjanMattei, CesarBatchu, RameshMatter, KarlBattista, SabrinaMattioli-Belmonte, MonicaBattistelli, CeciliaMattoscio, DomenicoBauersachs, StefanMattout, AnnaBaumstark-Khan, ChristaMatucci-Cerinic, MarcoBayik, DefneMatulic, MajaBayin, N. SumruMaturana, Andrés DanielBazil, JasonMatz-Soja, MadlenBeaujean, NathalieMauriz, José L.Beberok, ArturMavrogonatou, EleniBechstedt, SusanneMaxwell, Jessie R.Becker, JürgenMayanil, ChandraBecker, ThereseMazars, ChristianBeckett, EmmaMazat, Jean-PierreBedia, CarmenMazur-Bialy, Agnieszka IrenaBeemon, KarenMazzini, StefaniaBeffagna, GiorgiaMbalaviele, GabrielBehringer, ErikMccall, KellyBeineke, AndreasMcCarthy, JohnBejarano, IgnacioMcDaneld, Tara G.Bekeschus, SanderMcDermott, GerryBel’skaya, LyudmilaMcDermott, SuzanneBelardinelli, Juan ManuelMcDermott, Suzanne M.Belfiore, AntoninoMcDonagh, BrianBelizario, José ErnestoMcFarlane, Heather E.Bellahcène, AkeilaMcGettrick, H.M.Bello, MartinianoMcKay, BrianBeltrami, Antonio PaoloMcKenzie, DebbieBencivenga, StefanoMckenzie, MathewBen-David, YaacovMcKinney-Freeman, ShannonBen-Dov, Iddo Z.McKinnon, Katherine M.Benedetti, ElisabettaMcloughlin, PaulBenz, KorbinianMcMillin, MatthewBenzeroual, Kenza E.McPeek, AshleyBenzoni, PatriziaMears, JasonBerenyiova, AndreaMeccariello, RosariaBergamo, AlbertaMechulam, YvesBergeron, John J. M.Medalia, OhadBerleman, James E.Medina, DiegoBernardes, NunoMedvedev, AlexeiBernasconi, PiaMeerson, AriBernatík, OndřejMegraw, Timothy L.Berthod, FrançoisMegyeri, K.Berthold-Pluta, AnnaMehta, GauravBertoli, GloriaMei, FengBeverdam, AnnemiekMei, YangBeverly, Levi J.Meijerhof, TjarkoBewick, GuyMeinke, PeterBeyer, Eric C.Meissner, AnjaBhargava, AditiMekori, YosephBhargava, PavanMelcher, KarstenBhattacharjee, SonaliMelhem, HassanBhattacharya, SubhrajitMeli, AlbanoBhattacharya, SupriyoMelkani, GirishBhatti, Fazal-ur-RehmanMellor, HerryBhuiyan, Md. ShenuarinMeloni, GabrieleBiagi, MarcoMelzer, WernerBianchi Filho, CesarioMendes-Junior, CelsoBiasini, EmilianoMéndez Bolaina, EnriqueBiasini, EmilianoMenéndez, PabloBichet, Daniel G.Menendez, Sofia T.Bidarra, Sílvia J.Menezes, Gustavo B.Bidère, NicolasMennerich, DanielaBielle, FranckMenon, ManojBierhoff, HolgerMerdes, AndreasBilodeau, MarcMesuraca, MariaBinch, AbbieMetz, LuanneBiondi, AntonioMetzger, Herr PhilippBirchler, James A.Metzinger, LaurentBirge, Raymond B.Meunier, Jean-christopheBiro, MatéMeurer, Steffen KlausBitar, MaináMezzanzanica, DeliaBitler, BenjaminMezzasoma, LetiziaBizzaro, DeboraMiceli, VitaleBlack, Esther P.Michalak, KrystynaBlackmon, HeathMichalke, BernhardBlair, Geoger EricMichel, Martin ChristianBlaise, MickaelMichelle, PalmieriBlandino, GiovanniMichlewski, GracjanBlank, UlrichMielcarek, MichalBlankesteijn, MatthijsMiele, Adriana EricaBlažek, RadimMihály, AndrásBlazquez, Ana-BelenMika, DelphineBlind, RaymondMikala, GaborBliven, Spencer E.Mikelis, ConstantinosBłyszczuk, PrzemysławMikula, MarioBobek, VladimirMilardi, DaniloBobis-Wozowicz, SylwiaMilcarek, ChristineBoda, EnricaMildner, MichaelBode, Robert F.Milenkovic, Vladimir M.Bodea, LiviuMiles, Wayne OwenBoehme, Karen A.Miller, IngridBoerma, MarjanMiller, John H.Bogacka, IwonaMiller, JustinBoggon, TitusMiller, Robert H.Bogner, BarbaraMilovanovic, DragomirBoichuk, SergeiMimaki, YoshihiroBoink, Gerard JJMimche, Patrice NsangouBoissonneault, GuylainMimura, JunseiBoisvert, Francois-MichelMinami, YoichiBolanos-Garcia, Victor MMinden, Jonathan S.Boldizsár, FerencMinguet, Eugenio G.Bologna, RonellMinibayeva, FaridaBoltze, JohannesMiniero, Daniela ValeriaBonacci, MartínMione, Maria CaterinaBonanno, ElenaMiquel, AdroverBond, Heather M.Miragoli, MicheleBond, MarkMiranda, CristobalBondy, Stephen C.Mirzayans, RazmikBonsi, LauraMisasi, RobertaBoomer, Jonathan S.Missaglia, SaraBopegamage, ShubhadaMitkin, Nikita A.Bordonaro, MichaelMitra, KatsuriBorecki, MichalMitroulis, IoannisBorghi, NicolasMitsiadis, Thimios A.Borghini, AndreaMiyagoe-Suzuki, YukoBorodinsky, Laura N.Miyake, SachikoBoros, LászlóMiyamoto, KojiBorovac, Josip A.Miyamoto, ToshinobuBorriello, AdrianaMiyashita, ToshiyukiBorsani, ElisaMiyata, YasuyoshiBosch, MartaMiyata, YoshihikoBosch, Megan E.Mizukami, TakuoBose, DebojitMizushima, NoboruBottani, EmanuelaMlejnek, PetrBotti, GerardoMlera, LuwanikaBoualem, AdnaneModhiran, NaphakBoublikova, LudmilaMogielnicki, AndrzejBoucheix, ClaudeMoha Ou Maati, HamidBoudon, JulienMohamed, TamerBouhassira, EricMohr, AndreaBoulant, SteeveMolassiotis, AthanassiosBouley, RichardMoldzio, RudolfBourmeyster, NicolasMolenaar, Remco J.Bouschet, TristanMolinier, JeanBoutin, Jean A.Mollen, Kevin P.Bowerman, MelissaMoncharmont, BrunoBowyer, GeorginaMonchaud, DavidBoya, PatriciaMonferran, SylvieBoyman, OnurMongiat, MaurizioBozidis, PetrosMongin, AlexanderBozzaro, SalvatoreMonk, Pete N.Bracco, EnricoMonsalve, MariaBradbury, AllisonMontag, JudithBradshaw, NicholasMontagne, KevinBragazzi, Nicola LuigiMontalvo-Rodríguez, RafaelBrajušković, GMontana, GiovannaBrakebusch, CordMontanaro, LorenzoBramham, CliveMontarolo, FrancescaBranca, Jacopo Junio ValerioMonteleone, IvanBrand, KorbinianMontesinos, M. CarmenBrand, ThomasMontiel-Duarte, CristinaBrandenburg, Lars OveMontpetit, BenBrandt, RolandMooers, BlaineBrault, Jeffrey J.Moon, Il SooBraun, RalfMoon, Jong-SeokBraun, RobertMoore, Roger A.Braun, ThorstenMorales-González, José AntonioBrehm, PaulMoran, OscarBreitbart, HaimMorandi, FabioBreitkopf-Heinlein, KatjaMorandi, LucaBreloy, IsabelleMorbidelli, LuciaBrennan, KieranMoreaux, JeromeBresciani, ElenaMoreira, Ana CarolinaBresson-Bepoldin, LaurenceMoreira, DanieleBriata, PaolaMorena, FrancescoBrickner, JasonMoreno-Vinasco, LilianaBrigelius-Flohe, ReginaMorfini, GerardoBrindley, MelindaMorgado-Diaz, JoseBrini, MarisaMorgan, MichaelBrizuela, LeyreMori, GiorgiaBrizzi, Maria FeliceMoriscot, AnselmoBroaddus, Virginia Courtney F.Morita, NaokiBrodehl, AndreasMoriyama, HideakiBroer, StefanMoriyasu, YujiBros, MatthiasMorley, Simon J.Bross, PeterMoro, LoredanaBrotto, MarcoMorrison, Vicky LouiseBroude, EugeniaMosca, EttoreBrown, Amanda M.Mosqueira, MatiasBrown, Keith WMoss, WalterBrumeanu, Teodor-DoruMotrán, Claudia CristinaBrune, WolframMotrich, Rubén DaríoBrunetti, DarioMou, YongchaoBrunner, StefanMoudjou, MohammedBrünnert, DanielaMoukhles, HakimaBruserud, ØysteinMounien, LourdesBrüstle, AnneMousa, Shaaban A.Bryantsev, AntonMrakovcic, MariaBrzóska, EdytaMu, QinghuiBucci, CeciliaMuceniece, RutaBuchan, RossMuchir, AntoineBuday, LaszloMucignat, CarlaBueno-Silva, BrunoMueller, ThomasBuentello-Volante, BeatrizMuhammad, Jibran SualehBuhman, KimberlyMuhle, PaulBukiya, Anna N.Mujtaba, ShirazBullens, Dominique M.A.Mukai, TomoyukiBuratta, SandraMukaida, NaofumiBuravkova, LudmilaMukherjee, AbirBurchi, GianlucaMukherjee, RajibBurdak-Rothkamm, SusanneMukherjee, SudipBurdo, Tricia H.Mukouyama, Yoh-sukeBurdon, TomMüller, JanisBurgess, Janette KayMuller, MiryamBurgstaller, JoergMüller, PatrickBurk, JaninaMüller, SebastianBuschbeck, MarcusMuller, SylvianeBusinaro, RitaMulthoff, GabrieleBussolati, BenedettaMünch, JanBustamante, ClaudiaMuñoz, EduardoBustos, Diego MartinMuñoz, Estela M.Bustos, Victor H.Muñoz, José J.Busuttil, Rita A.Muñoz, PilarBuszewicz, DanielMuñoz-Chápuli, RamónBuzdin, AntonMuntané, GerardCabiscol, ElisaMuntane, JordiCaccamo, DanielaMunz, BarbaraCaceres, SaraMuraca, MaurizioCadamuro, MassimilianoMuraki, KatsuhikoCagnin, StefanoMurata, KoichiCai, HoujianMurata, TakayukiCai, JiyangMuriel, PabloCai, QianMurooka, ThomasCairrão, ElisaMuroya, SusumuCaivano, AntonellaMuruganandan, ShanmugamCakouros, DimitriosMusani, VesnaCakstina, IneseMuscella, AntonellaCalado, ÂngeloMusch, MarkCalado, Cecília R.C.Musti, Anna MariaCaldwell, Ruth B.Musumeci, GiuseppeCalì, BiancaMuthusamy, NagendranCalì, TitoMutti, LucianoCalinescu, AlexandraMyant, Kevin B.Call, Jarrod A.Mylonis, IliasCallaini, GiulianoMyre, MichaelCalvo Polanco, Maria MonicaNadel, SimonCalvo, Ana CristinaNadif Kasri, NaelCalzia, EnricoNagata, YosukeCamell, ChristinaNagy, NadineCameron, AngusNagy, TamásCameron, DonaldNahi, HarethCamins, AntoniNairz, ManfredCampanale, Joseph P.Naisbitt, Dean J.Campesi, IlariaNajafova, ZeynabCampos, CatarinaNajimi, MustaphaCampos, EricNakae, JunCampos, ManuelNakagawa, TakayukiCampos, Samuel K.Nakagawa, TakuroCampos-Toimil, ManuelNakagomi, TakayukiCamps, CarlosNakahama, Ken-ichiCampsteijn, CoenNakamura, NobuhiroCancemi, PatriziaNakamura, TsukasaCandela, Diaz-CanestroNakano, TakanariCanfield, ScottNakano, ToshiakiCanonico, BarbaraNakashima, YoshikiCantile, MonicaNakaya, NaokiCao, XuNakayama, KohCapitanio, NazzarenoNakayama, YujiCappelletti, VeraNakazawa, MitsuruCappello, ValentinaNakchbandi, InaamCarabineiro, SoniaNam, Hyun-JooCaraglia, MicheleNam, Ju-OckCarballo, IriaNamgaladze, DmitryCarboni, LuciaNan, HongmeiCardile, VeneraNanba, EijiCardinale, VincenzoNanbo, AsukaCardoso, Claudia R. L.Nanduri, RavikanthCardozo, Christopher P.Nanjareddy, KalpanaCarelli, StephanaNanni, LorisCaretti, AnnaNanoff, ChristianCarini, RitaNanou, AfroditiCarlberg, CarstenNapierala, MarekCarlin, Cathleen R.Napoli, EleonoraCarlisi, DanielaNappi, LuciaCarlos, Ana RitaNardelli, CarmelaCarmona, JuNarváez, ManuelCarneiro, Sueli Coelho Da SilvaNasarre, PatrickCarobbio, StefaniaNass, NorbertCaroleo, Maria CristinaNassar, Zeyad D.Carotenuto, MarcoNasser, MCarotti, SimoneNatarajan, Gayathri KrishnamoorthyCarpenter, RichardNauli, SuryaCarr, Carolyn A.Navedo, Manuel F.Carracedo, ArkaitzNawaz, MuhammadCarré, ClémentNaya, FranciscoCarroll, Michael C.Negishi, TakayukiCarroll, ThomasNegoda, AlexanderCartland, SianNegroiu, GabrielaCarulli, LuciaNeppl, Ronald LCarullo, GabrieleNervi, ClaraCaruso Bavisotto, CelesteNeumann, BrentCaruso, GiuseppeNeureiter, DanielCaruso, SalvatoreNew, ElizabethCarvalho, PedroNewsome, Timothy P.Carver, ChaseNewton, PhillipCasamassimi, AmeliaNgezahayo, AnacletCascione, MariafrancescaNguyen Phuoc, LongCashman, NeilNguyen, Chi HuuCasola, AntonellaNguyen, Khue VuCaspari, ThomasNguyen, VincentCassone, VincentNickerson, Jeffrey A.Castelblanco, EsmeraldaNicolás, Francisco E.Castellón, Enrique ANicolazzo, ChiaraCastillo-Gonzalez, Claudia MarcelaNicoletti, VincenzoCastorena-Torres, FabiolaNicolia, AlessandroCastoria, GabriellaNicolini, AndreaCastorina, AlessandroNicolini, GiuseppinaCastriconi, RobertaNicu, Elena A.Castro, Fidel OvidioNie, DaotaiCastro, MauroNiederberger, EllenCastrucci, Ana MariaNiemira, MagdalenaCato, Andrew C. B.Niepmann, MichaelCaton, PaulNiewiadomski, PawełCauffiez, ChristelleNighot, PrashantCauli, OmarNijnik, AnastasiaCavallaro, UgoNikiforov, MikhailCavazzoni, AndreaNikolaev, AntonCelia-Terrassa, ToniNikolaev, ViacheslavCenci, GiovanniNiland, StephanCeranowicz, PiotrNishida, KenseiCerdà, JoanNishihara, HidenoriCermak, LukasNishimura, NoriyukiČerná, MarieNishioka, ShintaCestmir, AltanerNishita, MichiruChachami, GeorgiaNishiyama, AkikoChagraoui, AbdeslamNissen, Peter H.Chai, WengangNistala, RaviChakrabarti, PriyadarshiniNiu, Chi-ChienChakraborty, SayanNivison-Smith, LisaChalah, Moussa AntoineNixon, Daniel W.Challa, DilipNiyazov, Dmitriy M.Challagundla, KishoreNjar, Vincent C.O.Chami, MouniaNjus, DavidChan, Ben C. L.Noda, NobuoChan, ClementNoguchi, Constance TomChan, David WaiNoguera, Nélida InésChan, JulieNohe, AnjaChan, Kuan RongNomura, SeitaroChan, Vera Sau-FongNor Eddine, SounniChandra, DhyanNoren Hooten, NicoleChang, Chuang-RungNorman, KennethChang, Hsueh-WeiNotara, MariaChang, Hui-YunNotaras, Michael J.Chang, Kuo-HsuanNovák, JanChang, MaylandNovío, SilviaChang, Nai-JenNowak, MarekChang, Shun-FuNumakawa, TadahiroChang, Tsung-HsienNúñez, José IgnacioChang, Wen-ChiNuytemans, KarenChang, Yuan-YenNylander, KarinChantry, AndrewNyström, AlexanderChao, Chia-TerO’Bryan, MoiraChao, Wei-TingO’Day, DantonChao, Yin XiaO’Neill, EricChapel, AlainO’Reilly, StevenChaptal, VincentO’Rourke, JamieChar, Hyuk-JinO’Rourke, Robert W.Charlet-Berguerand, NicholasO’Sullivan, MaryChatgilialoglu, ChryssostomosObermayr, EvaChatterjee, AniruddhaOchayon, David ElihaiChatterjee, IshitaOchoa-Reparaz, JavierChaussain, CatherineOcker, MatthiasChaves-Martinez, Felipe JavierOda, KatsutoshiChecler, FredericOddo, SalvotoreChen, ChiOdenthal, MargareteChen, Chung-HwanOdou, Marie FrançoiseChen, Chung-MingOfir, RivkaChen, Chun-JungOgawa, HidehikoChen, Georgia ZhuoOgórek, RafałChen, GuanOh, JaegyunChen, Guang-ChaoOhe, KenjiChen, GuoxunOhe, KenjiChen, LinOhl, KimChen, LiyeOhmura, KoichiroChen, MengOhnishi, MasatoshiChen, Miao-HsuehOhno, Shin-ichiroChen, MinOhta, YukoChen, PeiwenOhya, SusumuChen, Pei-WenOikawa, TsunekazuChen, QiOishi, AkioChen, Shuen-EiOka, ChioChen, ShukunOka, TakujiChen, ShuyangOkada, HideshiChen, Szu-yuanOkada, MasashiChen, TaipingOkajima, HideakiChen, WeiqinOkamoto, TakayukiChen, Wei-YuOkano, HideyukiChen, Wen-YingOkegawa, TakatsuguChen, XiaoyanOk-Seon, KwonChen, XiaozhuoOku, MasahideChen, XingOlczyk, PawełChen, XiupingOliart-Ros, Rosa MaríaChen, XunOliva, JoanChen, YehOliveira, Paulo J.Chen, Yei-TsungOlson, MichaelChen, Yi-JenOlson, Thomas L.Chen, YiliangOltra, ElisaChen, Yi-WenOmar, Syed HarisChen, Yu-ChihOndrej, VladanChen, ZhongOng, Sang BingCheng, ChunmingOnisto, MaurizioCheng, DeOnori, PaoloCheng, HailiOoi, JoshuaCheng, Heung-ChinOosting, JanCheng, Hung-ChiOpaliński, ŁukaszCheng, Juei-TangOpazo, CarlosCheng, XiangOpstad, Trine B.Cheng, XiaodongOrlov, SergeiCheon, Choong-illOrnelles, DavidChermnykh, ElinaOrsolic, NadaChern, EdwardOrsucci, DanieleCherry, JonathanOrthmann-Murphy, JenniferCheshenko, NataliaOrtiz, J. BryceCheungpasitporn, WisitOry, StéphaneChevalier, FrançoisOrzechowski, ArkadiuszChevret, EdithOsaki, MitsuhikoChew, Li-JinOshima, ShigeruChi, YulingOsman, AbdimajidChi, Yung-WeiOsna, NataliaChiabrando, Gustavo AlbertoOsol, GeorgeChiariello, MarioOsório, DanielChichger, HavoviOster, HenrikChilders, Delma SusanOstojic, SergejChin, Young-wonOstrom, RennoldsChinen, Ludmilla Thomé DomingosOsuna, AntonioChini, Eduardo N.Othumpangat, SreekumarChiocchetti, AnnalisaOtlewski, JacekChiou, Shih-HwaOtsuka, YuzuruChirumbolo, SalvatoreOttmann, MichèleChitraju, ChandramohanO-Uchi, JinChlichlia, KaterinaOussalah, AbderrahimCho, Dong-WooÖzdinler, P. HandeCho, HongsikOzretić, PetarChobot, VladimírPacak, ChristinaChoi, Jae SuePacal, LukasChoi, Kyeong SookPacheco, NeithChoi, YoukyungPacia, Marta Z.Chorianopoulou, Styliani N.Padilla-Benavides, TeresitaChou, Chung-LinPae, Eung-KwonChoudhary, SanjeevPaes, MarcianoChoudhuri, AvikPage, MelissaChoyke, Peter L.Paggetti, JérômeChrist, BrunoPagliara, PatriziaChrist, TorstenPaik, JihyeChristensen, Lars P.Pal, DebjaniChristodoulou, Maria-IoannaPal, KasturiChruszcz, MaksymilianPalani, ChitraChtarbanova, StanislavaPalaniappan, MurugesanChu, XianpingPalazón, AsísChuang, Show-MeiPalikaras, KonstantinosChubanov, VladimirPalladini, GiuseppinaChueh, Pin JuPalma Sircili, MarceloChuffa, Luiz GustavoPalomo, ValleChun, Kyung-SooPalumbo, CarlaChung, Byung MinPalumbo, PaolaChung, Jong HoonPalumbo, PasqualeChuntao, ZhaoPaluszczak, JarosławCiaramella, AngeloPalviainen, MariCiccarelli, RenataPan, JingCicero, ArrigoPan, MiaoCimini, DanielePanaro, Maria AntoniettaCimmino, GiovanniPanch, SandhyaCinque, BenedettaPandey, Ganshyam N.Cioffi, MarceloPandhi, AbhiCiofi-Baffoni, SimonePaneru, BamCione, ErikaPang, JinjiangCipak, LubosPannaccione, AnnaCippitelli, MarcoPanov, KonstantinCiribilli, YariPaone, AlessioCiuffreda, LudovicaPapaccio, GianpaoloClanchy, FelixPapadopoulos, PetrosClarke, Ronald JamesPapazafiropoulou, AthanasiaClausen, Anders R.Pappas, DimitriClawson, Gary A.Paradowska-Gorycka, AgnieszkaClere, NicolasParashar, DeepakCoccia, ElianaPardali, EvangeliaCock, CharlesPardigon, NathalieCohen, SarahParedes-Gamero, Edgar JulianCohen, SethParekh, AnantCoirault, CatherineParent, AudreyColeman, PaulParent, CaroleColitti, MonicaParent, RomainColl, RebeccaParikesit, Arli AdityaCollins, JenniferPark, Chan YoungCollins, Michelle M.Park, Hyun WooCollura, AdaPark, Jong KookColnaghi, LucaPark, Joo-CheolColombi, MarinaPark, Kyung-soonColombo, GiorgioPark, Sung-GyooColpitts, Che C.Park-min, Kyung HyunColuccia, MauroParlato, RosannaColumbano, AmedeoParnetti, LucillaComer, Jason E.Parola, MaurizioComi, CristoforoParoni, MoiraCommisso, MauroPascal, Steven M.Compton, AlexPasquinelli, GianandreaConde, CarlosPastores, Gregory M.Condello, MariaPatel, BhargavCondon, CiaránPatel, Daxesh P.Confalonieri, MarcoPaterlini-Bréchot, PatriziaConigliaro, AlicePatini, RomeoConover, Cheryl A.Patrie, Steven MatthewConsolo, UgoPatrone, CesareConte, CarmelaPattappa, GirishConti, HeatherPatterson, JenniferConti, PioPaul, FriedemannContreras, OsvaldoPaul, PercoContreras, Rubén G.Pautz, AndreaConway, Daniel E.Pavelić, KrešimirCook, LeahPavlik, EdwardCool, Robbert H.Pavlov, Chavdar S.Coombs, Kevin MPavlov, EvgenyCoppini, RaffaelePawar, SnehalataCoppotelli, GiuseppePawelek, JohnCorazzari, MarcoPearen, MichaelCorbet, CyrilPears, CatherineCordaro, MarikaPeart, JasonCordenonsi, MichelangeloPecho Vrieseling, ElineCordo Russo, Rosalia I.Pedemonte, NicolettaCorella, DoloresPedersen, Per AmstrupCorey, EvaPedeux, RémyCorfield, Anthony P.Peeples, EricCoria Ortega, RobertoPejler, GunnarCormio, AntonellaPellegrinelli, VanessaCornell, RobertPena, Maria MarjoretteCorrêa, Camila RenataPende, MarioCorreia-de-Sá, PauloPendin, DianaCortés, Kamila CaraballoPenke, BotondCortesi, LauraPenning, LouisCorti, FedericoPentimalli, FrancescaCorvalan, AlejandroPenzo, MariannaCorvigno, SaraPeppelenbosch, M.P.Costa, JuliaPeraldo-Neia, CaterinaCosta, VivianaPerales, JoseCosta-Almeida, RaquelPerego, CarlaCosta-Mallen, PaolaPereira Ferrer, ValeriaCostanza, MassimoPeretto, Paolo MarcelloCotelle, PhilippePérez Fernández, JavierCoulombe, PierrePérez-Campo, Flor MaríaCoulon, StéphanePérez-Méndez, ÓscarCourtois, GillesPerez-Perez, Maria EstherCoutant, FrédéricPérez-Sánchez, CarlosCouvineau, AlainPerez-Siles, GonzaloCraft, April M.Pergament, EugeneCrawford, Bryan D.Periyasamy, PalsamyCrawford, LindseyPerks, ClaireCrawley, AngelaPerl, AndrasCrespo, PieroPerni, MicheleCresteil, ThierryPernisová, MarkétaCrispin, Jose CPero, RaffaelaCrnkovic, SlavenPeronnet, FrederiqueCronauer, Marcus V.Perrone-Bizzozero, Nora I.Crook, Zachary R.Persechini, AnthonyCros, JérômePersson, EmmaCross, Frederick R.Perucha, EsperanzaCross, MichaelPeruzzi, FrancescaCross, MichaelPešić, MilicaCrudden, CaitrinPestov, DimitriCrus-Topete, DianaPetan, ToniCsiszar, AgnesPetit, Patrice X.Csont, TamásPetrella, AntonelloCuevas-Romero, EstelaPetrillo, SaraCui, JiuweiPetroll, MatthewCui, TiantianPetroni, MarialauraCulot, MaximePetropoulos, IsabelleCummings, BrianPetrosyan, ArmenCurtale, GraziellaPetrou, Athinoula L.Cusanelli, EmilioPetrov, Alexey M.Cyganek, LukasPetzold, TobiasCytlak, UrszulaPeulen, OlivierCzikora, IstvanPeviani, MarcoCzyżewski, KrzysztofPEYRON, Jean-FrançoisD Prestwich, Robin JPhilipp, ManfredD’ Adda di Fagagna, FabrizioPhilips, NeenaD’adamo, StefaniaPhillips, LindaD’Amato, AlfonsinaPhillips, Nicole R.D’Ambrosi, NadiaPhillips, WilliamD’Amelio, StefanoPhilpott, MichaelD’Antonio, MatteoPiancatelli, DanielaD’Onofrio, NunziaPiazza, FrancescoD’Orléans-Juste, PédroPichon, MaximeDa Silva, M. L. AlvesPicone, DeliaDaci, ArmondPidoux, GuillaumeDagda, Ruben K.Piedra, PedroDai, Chia-YenPierno, SabataDalamaga, MariaPierzchalski, PiotrDalhaimer, PaulPignatti, PatriziaDalin, Martin G.Piiper, AlbrechtDallmeier, KaiPineau, PascalDamdimopoulou, PauliinaPineda, MiguelDamia, GiovannaPinho, VanessaDamiani, DanielaPinkas, AdiDan, Yock YoungPinto, MafaldaDandawate, PrasadPinto, MilenaDanelli, LucaPinzani, PamelaDani, ChristianPioner, Josè ManuelDaniel, JulietPiosik, JacekDaniele, AuroraPiovan, ErichDanisovic, LubosPiperno, AnnaDanti, SerenaPires, Bruno R. B.Dao Thi, Viet LoanPirkmajer, SergejDarling, AprilPirro, StefanoDas, AmitavaPisani, LucianaDas, AninditaPislariu, CatalinaDas, GokulPistello, MauroDas, SiddharthaPitt, SamanthaDas, ViswanathPitto, LetiziaDasgupta, SantanuPiwowar, AgnieszkaDash, BirajaPizzato, MassimoDatta, PoulamiPizzimenti, StefaniaDaubon, ThomasPizzo, ElioDave, Kunjan R.Pizzolanti, GiuseppeDave, SandeepPlant, Timothy D.David, RobertPlatta, HaraldDavis, A. SallyPlaza-Díaz, JulioDavis, Richard E.Plaza-Sirvent, CarlosDawson-Scully, KenPlaza-Zabala, AinhoaDe Andrade, MarizaPlemel, JasonDe Andrea, MarcoPlotnikov, Egor Y.De Assis, Leonardo V MPluta, RyszardDe Berardis, DomenicoPobbati, AjaybabuDe Berardis, DomenicoPócsi, IstvánDe Berardis, DomenicoPodar, KlausDe Brevern, Alexandre G.Poeggeler, BurkhardDe Chiara, LetiziaPoelmann, RobertDe Curtis, IvanPoggi, AlessandroDe Falco, ElenaPohjoismäki, Jaakko L. O.De Felice, BrunaPohl, ChristianDe Felici, MassimoPohl, SebastianDe Francesco, FrancescoPohlkamp, TheresaDe Geest, BartPoirier, Guy G.De Gonzalo, DavidPoitelon, YannickDe La Fuente, HortensiaPóka, RóbertDe La Fuente, MónicaPolacek, NorbertDe Luca, AntonellaPolak, Marta EwaDe Lucca Camargo, LiviaPolańczyk, AndrzejDe Martino, AndreaPolge, CécileDe Masi, RobertoPoli, AlessandroDe Meyer, TimPolishchuk, RomanDe Nijs, LaurencePoltronieri, PalmiroDe Nola, RosalbaPoluektova, Larisade Noronha, CarlosPompili, ElenaDe Palma, AnnaPonnusamy, Moorthy P.De Pinto, VitoPons, MiquelDe Rasmo, DomenicoPons, SebastianDe Robertis, MariangelaPonzoni, MircoDe Simone, Salvatore GiovanniPoór, MiklósDe Vita, AlessandroPop, Tudor LucianDe Vries, Antoine A.F.Popa-Wagner, AurelDe Waard, VivianPope, ChadDe Zio, DanielaPope, SimonDe, PradipPopeijus, Herman E.Dealy, Caroline N.Popper, Helmut H.Dean, FrankPorat, ZivDearborn, Donald C.Porebski, GrzegorzDecuypere, Jean-PaulPorokhovnik, LevDed, LukasPort, Sarah A.DeGrado, Timothy R.Porta, HelenaDegrassi, FrancescaPorter, GeorgeDehay, BenjaminPorter, RichardDel Fattore, AndreaPosey, Avery D.Del Fresno, CarlosPossik, EliteDel Porto, PaolaPotaczek, Daniel P.Delanoë-Ayari, HélènePotiron, VincentDella Ragione, FulvioPotokar, MajaDellambra, ElenaPoudel, BarunDelle Monache, SimonaPoudrier, JohanneDemarquoy, JeanPouliot, MarcDembska, Anna RenataPourcet, BenoîtDemer, LindaPowers, TedDemir, Ihsan EkinPozo Devoto, VictorioDeng, Win-PingPozzato, GabrieleDengler, FranziskaPozzobon, MichelaDeo, PermalPradotto, Luca GuglielmoDerenzini, MassimoPrange, ReinhildDerer, StefaniePrashar, SanjivDeschenes, MichaelPratsinis, HarrisDeshmukh, RupeshPredescu, DanDeshpande, ShayuPrêle, Cecilia M.Desprès, PhilippePrescott, Steven A.Dessauer, CarmenPreto, AnaDeugnier, Marie AngePrevis, Rebecca A.Deutsch, AlexanderPriestman, David A.Devaux, PatriciaPriglinger, EleniDevirgiliis, ChiaraPrivette Vinnedge, LisaDewitte, WalterProchazka, RadekDhaenens, MaartenProchownik, Edward V.Dhamija, SonamProsser, Robert ScottDhandayuthapani, SubramanianProszynski, TomekDharmawardhane, SuranganiePrudent, JulienDhungel, BijayPrudent, MarionDi Baldassarre, AngelaPrudovsky, IgorDi Blasio, LauraPruitt, KevinDi Caro, AntoninoPuca, AnnibaleDi Donato, MarziaPuca, LoredanaDi Girolamo, MariaPuchkova, Ludmila V.Di Liegro, ItaliaPuertas, Maria CarmenDi Maro, AntimoPufall, MilesDi Martino, EricaPujol, AïdaDi Matteo, AdelePulaski, LukaszDi Micco, RaffaellaPulgar, VictorDi Micco, SimonePurdy, JohnDi Renzo, Maria FlaviaPushchina, EvgeniyaDi Ruscio, AnnalisaPuthanveettil, SathyaDi Sansebastiano, Gian PietroPuthier, DenisDi Stasi, AntonioPuustinen, PietriDi Zazzo, ErikaPuvvula, Pavan KumarDíaz Mochón, Juan JoséQasaimeh, Mohammad A.Díaz-Guerra, MargaritaQasim, SaadDíaz-Moreno, IreneQian, HongDiccianni, MitchellQiao, HuanyuDidonna, AlessandroQie, YaqingDiefenbach, Russell J.Qin, HongminDiefenbacher, Markus E.Qiu, BinDierickx, PieterjanQiu, HuanDiermeier, SarahQu, PengDietrich, AlexanderQuan, Fu-ShiDietrich, PeterQue, JennyDijkstra, GerardQueiroga, FelisbinaDijkstra, Johannes M.Queralt, EthelDimova, ElitsaQuintana, MarcosDing, NingQuintarelli, ConcettaDing, QingbaoQuirós, Luis M.Dinh, Dzung H.Raab, Marc SteffenDissanayaka, WarunaRacine, TrinaDittmar, ThomasRadenkovic, MiroslavDivanovic, SenadRadic, MarcoDiviani, DarioRadner, FranzDivino-Filho, JosèRadošević-Stašić, BiserkaDixon, BrianRadu, Beatrice MihaelaDjavahery Mergny, MojganRadu, MihaiDludla, Phiwayinkosi V.Rafiei, HosseinDoekes, GertRagavan, MukundanDoering, TamaraRagni, EnricoDoig, CraigRagnini-Wilson, AntonellaDolan, Brian P.Ragusa, Maria AntoniettaDolzblasz, AlicjaRagusa, Michael J.Dolzhenko, Anton V.Rahal, OmarDombrowicz, DavidRahimi, FaridDombrowski, YvonneRahman, M. AzizurDonadel, GiuliaRahman, SafikurDonalisio, ManuelaRahmathulla, GazanfarDonati, GiacomoRai, PrakashDonato, RosarioRai, SumitDong, FengRaimondi, LaviniaDonizy, PiotrRaina, KomalDoody, GinaRaina, MedhaDöppner, Thorsten R.Rajnarayanan, RajendramDoroudgar, ShirinRakus, DariuszDos Santos, Reinaldo SousaRamaiahgari, Sreenivasa C.Dosil, MercedesRamalingam, NagendranDou, ZhixunRaman, DayanidhiDráber, PavelRamaswamy, BhuvaneswariDrabkin, HarryRamesh, SangeethaDraghici, SorinRamić, SnježanaDragomir, MihneaRamil, CarloDrakakaki, GeorgiaRamírez Sánchez, ManuelDrutovic, DavidRamirez, Giuseppe A.DS, GalileoRamírez-Bergeron, DianaDu Pré, BastiaanRamos Cabrer, PedroDu, MingRamos, YolandeDuan, ZhijunRampazzo, ElenaDuarte, FilipeRan, ChongzhaoDubash, TaronishRangachari, ManuDubey, RaghvendraRanieri, DaniloDubińska-Magiera, MagdaRanjan, KishuDubrova, YuriRantamaki, TomiDucza, EszterRanzato, EliaDuda, MałgorzataRao, VidhyaDudok, JeroenRapp, AlexanderDufrene, YvesRárová, LucieDuncan, Raymond ScottRasche, LeoDuncan, Robert KeithRashid, TamirDundr, MiroslavRašin, Mladen RokoDunlop, ElaineRatajewski, MarcinDunn, PeterRathinasabapathy, AnandharajanDupre, AudeRauch, Bernhard H.Duquette, PierreRauvala, HeikkiDurand, BénédicteRavanidis, StylianosDurnford, DionRavazzolo, RobertoDürrbach, AntoineRavegnini, GloriaDutour Sikirić, MajaRavnik-Glavač, MetkaDutta, ArijitRavuri, SudheerDworatzek, ElkeRay, RanjitDwyer-Nield, Lori D.Rayaprolu, SrutiDziegielewska, BarbaraRaymond, AndreaDzobo, KevinRazumilava, NataliyaEar, JasonReale, AnnaEarl, JulieRebbeck, RobynEbert, Allison D.Rebelo, SandraEbhardt, H. AlexanderRebelo, Sofia P.Eboigbodin, KevinReddy, Marpadga A.Ecelbarger, Carolyn A.Reddy, SakamuriEchevarria, MiriamReddy, Sudhir PuttyEckl, KatjaRedell, MicheleEckl, PeterRedondo-Muñoz, JavierEdemir, BayramReed, DamonEdgar, DavidReed, Miranda NicoleEdlich, FrankReese, Benjamin E.Eeckhoute, JérômeRegad, TarikEftekharpour, EftekharReggiori, FulvioEfthymios, MotakisRégnier-Vigouroux, AnneEgerszegi, IstvánRehmann, HolgerEhnert, SabrinaRehmann, HolgerEhrmann, MichaelReid, St PatrickEichhorn, PieterReiling, Jan H.Eid, NabilRein, TheoEiges, RachelReis, Celso A.Eijkelkamp, Niels N.Reis, Fernando MEisenberg, TobiasRelat, JoanaEisenmann, Kathryn M.Remaley, AlanEizaguirre, ChristopheRen, JunEkberg, JennyRenaudineau, YvesEl Bairi, KhalidResende, RosaEl Midaoui, AdilResnick, AndrewEldeeb, MohamedRezaei-Ghaleh, NasrollahEl-Hachem, NehmeRežen, TadejaElias, Maria CarolinaRhee, Je-KeunEliseeva, IrinaRiar, Amanjot KaurEljaafari, AssiaRibchester, RichardElliott, TimRicardo, SharonElmarakby, AhmedRice, AndrewElsner, MatthiasRice, Charles D.Emerson, Mark M.Richard, EmmanuelEmri, GabriellaRichard, MelissaEndersby, RaeleneRichards, Carl D.Endo, MakotoRiches-Suman, KirstenEngedal, NikolaiRichner, JustinEngel, StanislavRichter, KatrinEngelberth, JurgenRieder, LeilaEngelmann, IlkaRieg, TimoEnguita, FranciscoRiemann, MichaelEnyedi, PéterRiester, Scott M.Erdő, FranciskaRimer, MendellErenpreisa, JekaterinaRinnerthaler, MarkErgün, SüleymanRisbridger, GailEri, S. SrivatsanRivera, VictorErukainure, OchukoRivera, Víctor M.Escamez, SachaRivero, FranciscoEsclatine, AudreyRivero, Rosa MEskelinen, Eeva-LiisaRizos, HelenEskiw, Christopher H.Rizza, SalvatoreEsmaili, SaeedRobert, JacquesEspada, JesúsRoberts, Stephen K.Espejo, CarmenRobin, Jérôme D.Espel, EnricRobinson, CliveEsposito, FrancescoRobinson, DouglasEsser, Jennifer S.Robson, Matthew JamesEstrada-Gutierrez, GuadalupeRocha, SoniaEtienne-Manneville, SandrineRocha, SoniaEtxebeste, OierRocheleau, GhislainEubank, TimothyRochfort, KeithEvergren, EmmaRocks, OliverEvinger, MarianRodellar, ClementinaEyers, PatrickRodellar, JoseFabian, ZsoltRodenbeck, Stacey DineenFabris, LucaRodil, SandraFahrenkrog, BirtheRodin, Sergey A.Failla, Cristina M.Rodolfo, CarloFairchild, RobertRodríguez, AmaiaFairlie, DougRodriguez-Vargas, Jose M.Faitot, FrancoisRodriguez-Vita, JuanFalcone, GermanaRoduit, RaphaelFalzone, LucaRoelen, BernardFameli, NicolaRoelink, HenkFan, DapingRogasevskaia, TatianaFan, Guo-changRogelj, BorisFan, Guo-ChangRoger, JérômeFan, Hueng-ChuenRogers, HilaryFan, WeiweiRoh, JaesookFan, YiRoh, SanghoFandrey, JoachimRohlena, JakubFang, Evandro F.Rokavec, MatjazFang, JunnanRomanelli, Maria NovellaFang, QingmingRomani, PatriziaFang, XianjunRomano, MaurizioFantini, MassimoRomeo, MargaritaFaraone Mennella, Maria RosariaRomero, CarmenFarcasanu, Ileana C.Romero, DamianFarlik, MatthiasRomero, Marta R.Faron-Górecka, AgataRomero-Lorca, AliciaFarrar, Michael A.Romito, LuigiFarrell, RobertRoncal, CarmenFarrugia, BrookeRonchetti, SimonaFasolato, CristinaRondeau, PhilippeFaustino, RandolphRonnberg, ElinFaux, NoelRonza, PaoloFeakins, RogerRoosenboom, BrittFedorova, MariaRoot, MartinFedotova, Julia O.Roper, Randall J.Feichtinger, Rene GRoque, CláudioFeige, Jean-JacquesRosado, JuanFeliciello, AntonioRosati, JessicaFeng, WenkeRosato, AntonioFeng, YiRose, MichaelFenizia, FrancescaRosenberger, ChristianFereshtehnejad, Seyed-MohammadRosenmann, HannaFerguson, DavidRosenthal, Dean S.Ferguson, MatthewRosenzweig, DerekFerguson, StephenRosic, GvozdenFerini-Strambi, LuigiRoss, IanFeris, Edmond J.Rossa Jr, CarlosFernandes, HugoRossi, WaldemarFernandes, MónicaRossignol, JulienFernández De Mattos, SilviaRossner, PavelFernandez-Botran, RafaelRosso, NataliaFernández-Sanz, CeliaRossowska, JoannaFernandez-Zapico, M.D. Martin E.Rotblat, BarakFerrante, ClaudioRoth, MichaelFerraz-de-Souza, BrunoRottbauer, WolfgangFerreira, Andrea C. F.Rottmar, MarkusFerretti, ElisabettaRouault-Pierre, KevinFerri, Giovanni MariaRoudbaraki, MoradFerrigno, AndreaRouillé, YvesFerriz-Martínez, Roberto AugustoRounge, TrineFerroni, PatriziaRoux, KyleFestuccia, NicolaRowther, Farjana B.Fiandra, LuisaRoy, DeoduttaFiaschi, TaniaRoy, SoumenFigueira, Ana Carolina MiglioriniRoyle, Nicola J.Filella, XavierRozman, DamjanaFilep, János G.Rual, Jean-FrançoisFilipeanu, CatalinRucker, Edmund B.Filomeni, GiuseppeRückert, FelixFilosto, MassimilianoRudolf, EmilFinco, IsabellaRudolf, RuedigerFinelli, CarmineRudyk, OlenaFinkelman, Hagit EldarRuiz De Almodóvar, CarmenFiorio Pla, AlessandraRuiz-Cabello, JesusFiorucci, StefanoRurali, EricaFirestein, RonRus, Horea G.Fischer, AndreasRusmini, PaolaFisher, Kevin E.Russo, AnnapinaFisher-Wellman, KelseyRusso, AntonioFiszer, AgnieszkaRusso, Matteo A.Fitter, JörgRuusuvuori, PekkaFitzgerald, DavidRuvio, RaquelFlacher, VincentRyter, StefanFlaherty, KevinRyu, ChangseonFlamigni, FlavioRzepecki, RyszardFlamion, BrunoRzońca, EwaFlanagan, Eoin P.Saada, AnnFlavell, RobertSaadoun, SamiraFleischman, Angela G.Saba, Nakhle S.Fliedner, StephanieSabaawy, Hatem E.Flores, IgnacioSabol, MajaFoecking, MelanieSacchetti, BenedettoFogarty, GeraldSaccon, LeonardoFojtová, MiloslavaSaccone, ValentinaFolco, Hernan DiegoSack, UlrichFoley, Ann C.Sadkowski, TomaszFolini, MarcoSadowski, MartinFonseca-Alves, Carlos EduardoSaeki, KumikoFontanesi, FlaviaSaferding, VictoriaForini, Francesca S.Saha, AchintoFormisano, LuigiSaha, Amit KumarFornai, FrancescoSaha, Subbroto KumarForte, EleonoraSaha, SuryaForte, MaurizioSahara, NaruhikoFortunato, OrazioSaharinen, PipsaFortunel, NicolasSahni, Sanjeev K.Fouda, AbdelrahmanSahni, SumitFouquerel, EliseSaid, NeveenFournier, AntoineSaiki, ShinjiFowler, ChristieSaini, ShikhaFowler, Velia M.Sainlos, MatthieuFox, ToddSaitoh, KenjiFraineau, SylvainSaitoh, MasaoFranca Junior, Marcondes CavalcanteSakai, HiromichiFrancastel, ClaireSakai, TakahiroFrancis, HeatherSakai, TakaoFranco, OmarSakamuro, DaitokuFranco, PierfrancescoSakata, NaoakiFranco-Barraza, JanuszSala, AmbreFrange, PierreSala, GessicaFranzini, AncaSala, Lucia LaFreichel, MarcSala, RobertoFreitas, Vanessa MoraisSalazar, GloriaFrémont, StéphaneSalbert, GillesFrentiu, FrancescaSalem, AbdelhakimFreude, KristineSalgado, RobertoFriant, SylvieSalifoglou, AthanasiosFrič, JanSalinska, ElzbietaFrick, DavidSalmon, Jessica M.Frieden, MaudSalucci, SaraFrietze, SethSaluk-Bijak, JoannaFriis-Hansen, LennartSamant, Rajeev S.Frippiat, Jean-PolSamant, Rajeev S.Frischauf, IreneSamarakoon, RohanFritsch, Ralph M.Samarut, EricFrossi, BarbaraSamhan-Arias, AlejandroFu, BeideSamir, ParimalFu, QinSampson, Timothy R.Fu, Sidney W.Samsonov, AlexeyFu, XingSamuels, IvyFukuhara, AtsunoriSanak, MarekFukumitsu, KenSanchez, AnaFunaro, AdaSanchez-Alcazar, JoseFunato, NorikoSánchez-Duffhues, GonzaloFurukawa, YoshikoSánchez-Madrid, FranciscoFuruya, HidekiSanchez-Martinez, AlvaroFutrega, KathrynSánchez-Pérez, Ana MaríaFyodorov, DmitrySander, VeronikaG Gamage, DilaniSang, YongmingGaber, TimoSangadala, SreedharaGache, VincentSanna, DariaGad, AnnicaSantacroce, LuigiGadalla, Shahinaz M.Santaguida, StefanoGadani, SachinSantamaria, DavidGadeau, Alain-PierreSantarelli, AndreaGafurov, MaratSantolla, Maria FrancescaGaglia, Marta MariaSantoro, RaffaellaGagliano, NicolettaSantos, Ana PaulaGagliano, TeresaSantos, JerranGagliardi, Paolo ArmandoSantos, Joao MiguelGagneux, PascalSantos, Jose MariaGaida, MatthiasSantucci, AnnalisaGál, PeterSantulli, GaetanoGalardi, SilviaSanz Luque, EmanuelGalbiati, SilviaSanz, PascualGalgani, MarioSaotome, MasaoGaliacy, StéphaneSapudom, JiranuwatGaligniana, Mario D.Saraiva, NunoGalindo, AntonioSarasija, ShaarikaGalisteo Jr., Andres JimenezSarathy, Vanessa V.Gall Troselj, KoraljkaSaretzki, GabrieleGallay, PhilippeSaroussi, ShaiGalli, DanielaSase, AjinkyaGallitano-Mendel, AmeliaSato, MasamitsuGallo, AlessiaSato-Bigbee, CarmenGaluska, Sebastian P.Satoh, KiyotoshiGaluska, Sebastian PeterSattlegger, EvelynGalvagni, FedericoSattler, SusanneGalván, AuroraSaussez, SvenGalvao, IzabelaSavaskan, Nicolai E.Gálvez, JulioSavastano, SilviaGama-Carvalho, MargaridaSavica, RodolfoGanau, MarioSavoldo, BarbaraGanem, Neil J.Sawamura, NaoyaGanesan, Latha P.Sayer, JohnGanji, RakeshScafidi, JosephGanley, AustenScaglione, Kenneth MattGantier, MichaelScarfì, SoniaGao, BeiScarpa, SigfridoGao, BinScatena, RobertoGao, DingchengSchaaf, MarcelGao, MinSchaefer, MichaelGao, PeisongSchaeffel, FrankGao, ZheSchäffner, AntonGarbuglia, Anna RosaSchaft, NielsGarcia Perez, SamuelSchallmoser, KatharinaGarcía, GabrielaSchang, LuisGarcía-González, VíctorScharf, AndreaGarcia-Pardo, JavierScheibenbogen, CarmenGarcia-Robles, InmaculadaScheibye-Knudsen, MortenGarcía-Vicuña, RosarioScheller, Erica L.Garinis, GeorgeSchenone, SilviaGarousi, JavadScheu, StefanieGarrido-Ramos, ManuelSchierwagen, RobertGasparri, FabioSchildgen, OlivierGassler, NikolausSchilling, JoelGassman, NatalieSchirmer, EricGastaminza, PabloSchlachetzki, JohannesGattei, ValterSchlaepfer, Isabel RGatti, LauraSchlondorff, JohannesGaudieri, SilvanaSchlüter, Klaus-DieterGautam, PrsonSchmid, Johannes AlfredGauthier, NilsSchmitz, ThomasGavalas, AnthonySchmitz, UlfGaziano, RobertaSchmolke, MircoGazit, RoiSchnauß, JörgGazouli, MariaSchneider, Bernard LaurentGe, YubinSchneider-Maunoury, SylvieGebhardt, RolfSchöfer, ChristianGeisert, Rodney D.Scholich, KlausGeisler, FabianSchönberger, StefanGeisler-Lee, JaneSchonewille, MartijnGeißler, ChristinSchrul, BiancaGelissen, IngridSchueler, JuliaGendek-Kubiak, HannaSchuetz, AlexandraGenders, AmandaSchughart, KlausGeorgakilas, AlexandrosSchülke, StefanGeorge, SoniaSchulze-Tanzil, GundulaGeorgiadi, AnastasiaSchulz-Heddergott, RamonaGeorgiou, ChristosSchumacher, AnneGeorgopoulou, UraniaSchumacher, UdoGepner, YftachSchutte, StaceyGeraci, FabianaSchwalbe, HaraldGerber, AndréSchwartz, АntonGerhardt, ChristophSchweisguth, FrançoisGerlitz, GabiSchweizer, Felix E.Germain, LucieSchwind, SebastianGermain, PierreScicchitano, PietroGermano, GiovanniScioscia, CrescenzioGerriets, ValerieSciumè, GiuseppeGershon, TimothyScorziello, AntonellaGerst, Jeffrey E.Scott, Mark G.H.Gewirtz, DavidScotti, MarcusGhag, GauravScotto D’Abusco, AnnaGhezzi, DanieleSeano, GiorgioGhiggeri, Gian MarcoSebastian, AimyGHILA, LuizaSebastian, CarlosGhinassi, BarbaraSecondo, AgneseGhirga, FrancescaSeddon, JenniferGhislat, GhitaSedic, MirelaGhosh, SantoshSee, ViolaineGiacomelli, ChiaraSeeliger, ClaudineGiacomello, EmilianaSeeman, Mary V.Giakountis, AntonisSefton, MichaelGiammanco, MarcoSegatto, MarcoGiannakouros, ThomasSeguin, Cheryle A.Giannatasio, SergioSeki, NaohikoGieras, AnnaSellami, AkremGiet, RegisSeluanov, AndreiGiglia-Mari, AmbraSemerci, NihanGil, Cristiane DamasSemina, Ekaterina V.Gilch, SabineSenpuku, HidenobuGiles, CoreySeok, Seung HyeokGill, Pritmohinder S.Sergi, DomenicoGilloteaux, JacquesSerini, GuidoGillum, Matthew P.Serino, GiovannaGil-Martín, EmilioSerpa, JacintaGilmer, JohnSerrano, MercedesGiordano, CarlaSerre-Beinier, VéroniqueGiordano, SilviaSeth, PankajGiovannetti, ElisaSethi, GautamGiovannoni, RobertoSethmann, IngoGiovarelli, MatteoSeubert, John MGiovarelli, MirellaShah, KhyatiGirao, HenriqueShah, RuchiGiri, HemantShaikhali, JehadGirnita, LeonardShaish, AvivGiron, Maria CeciliaShankar, EswarGirstun, AgnieszkaShanmugam, MalaGissen, PaulShanmugam, MuruganandanGiuffrida, RosarioSharif, JafarGiugno, RosalbaSharma, AlokGiusti, IlariaSharma, Amar DeepGkretsi, VasilikiSharma, ArishyaGłąbska, DominikaSharma, DipaliGnanasundram, Sivakumar VadivelSharma, GeetanjaliGo, Gwang-WoongSharma, NeeruGoberdhan, DeborahShaul, Yoav D.Goda, KatalinShavandi, AminGodos, JustynaShaw, SubrataGodsel, Lisa MarieShehadeh, Lina A.Goel, Peeyush N.Sheikh, NadeemGoglia, FernandoSheikhbahaei, ShahriarGogvadze, VladimirSheldrake, HelenGoh, Bey-HingShen, GarryGokare, PrashanthShen, HaihongGolanov, Eugene V.Shen, Han-MingGold, MatthewShen, XingguiGoldberg, AlfredSheng, YueGolden, AaronShenolikar, ShirishGoldman, JamesSherman, PhilipGołembiowska, KrystynaSheth, SandeepGolinelli, Marie-PierreSheval, EugeneGoljanek-Whysall, KasiaShevelyov, YuriGoljanek-Whysall, KatarzynaShi, HaifeiGolstein, PierreShi, LeiGolstov, AlexeyShi, WenGolubovskaya, VitaShi, YanGomer, RichardShibata, HiroshiGomez, LudovicShibata, YasushiGomez, NidiaShieh, Jeng-jerGomez-Lazaro, MariaShigeru, HishinumaGomez-Roman, NatividadShiina, MarisaGómez-Sánchez, RubénShim, Joon W.Goncalves, OlivierShim, Joong SupGonçalves, VâniaShimodaira, ShigetakaGonzález Granado, José MaríaShin, Chong HyunGonzalez, AntonioShin, Dong-MyungGonzalez, RogelioShin, KwanwooGonzález, SegundoShin, Vivian YvonneGonzález-Álvaro, IsidoroShingu, TakashiGonzalez-Bulnes, AntonioShinoka, ToshiharuGonzález-Navarro, HerminiaShintani, YasushiGoodier, Martin R.Shioda, TatsuoGoodman, AlanShirakami, YoheiGordeeva, OlgaShirato, KenGorio, AlfredoShirinian, MargretGoris, AnShirokikh, Nikolay E.Gorrini, ChiaraShivapurkar, NarayanGoswami, AnandShnyder, SteveGoswami, AnweshaShoji, IkuoGothilf, YoavShoshan, MariaGouws, ChrisnaShtutman, MichaelGov, NirShu, SherryGowran, AoifeShukla, HemGozal, EvelyneShukla, SiddharthGrabinska, KarionaSibson, David RossGrabowska, Anna M.Siddharthan, VenkatramanGraça, LuísSiddiqui, ImranGraeve, LutzSidorenko, Viktoriya S.Gräf, RalphSidransky, EllenGraier, WolfgangSiebenhaar, FrankGramignoli, RobertoSiebermair, JohannesGrand, Roger J.Siegel, Sue RutherfordGrande, AlexisSiegmund, BrittaGrandemange, StéphanieSierra-Campos, ErickGrange, CristinaSigal, MichaelGranot, DavidSigala, SandraGrant, MichaelSignorile, AnnaGrãos, MárioSikora, MariuszGrau, VeronikaSikorski, AleksanderGray, AndrewSills, E ScottGrayfer, LeonSilva, Adriana RibeiroGreene, Catherine M.Silva, AndreGreenlee-Wacker, MallarySilva, GustavoGreenwood, Michael T.Silva, Nuno A.Gregory, StephenSilva, RenatoGreiner, JohannesSilvan, UnaiGrela, PrzemysławSilvestri, IdaGrelet, SimonSimanshu, Dhirendra K.Grewal, Raji PaulSimeoli, RaffaeleGrewal, ThomasSimitzi, CharaGrifoni, DanielaSimoes, BrunoGrimaldi, GiovannaSimon, FelipeGrimm, MarcusSimon, Julianna C.Grinberg, DanielSimonetti, GiorgiaGrippo, Paul JŠimoník, OndřejGris, DenisSimpson, Benjamin S.Grizzi, FabioSinger, Mitchell H.Grodzik, MartaSingh, AnandGrolli, StefanoSingh, AnuragGrose, JulianneSingh, AnuragGroßhans, JörgSingh, BhagirathGrube, SusanneSingh, ChanpreetGruber, ChristianSingh, GurinderGrubić Kezele, TanjaSingh, HarinderGrunebaum, EyalSingh, ParamGrünewald, ThomasSingh, RajbirGrunow, BiankaSingh, RatnakarGruntman, Alisha M.Sinha, DebottamGrusch, MichaelSinha, KalyanGruss, OliverSinha, NiharikaGrützner, FrankSinha, SangitaGrzybowska, EwaSinning, ChristophGuang, ShouhongSipos, KatalinGudermann, TSirenko, OksanaGudey, Shyam KumarSiristatidis, CharalamposGudi, ViktoriaSitcheran, RaquelGuemez-Gamboa, AliciaSivakumar, SushamaGuerra, Alfredo J.Sivaprakasam, SathishGuerra, FloraSkendros, PanagiotisGuerra-Sá, RenataSkidmore, MarkGuerreiro, Joana FSkoko, John J.Guerriero, GiuliaSkonieczna, MagdalenaGuescini, MicheleSkonieczna-Żydecka, KarolinaGugnoni, MilaSkretas, GeorgiosGuha, Prajna P.Skripuletz, ThomasGuha, PrasunSkrzypski, MarekGuiamet, Juan-JoséSkubitz, Amy P. N.Guidi, SandraSkundric, Dusanka SGuillerey, CamilleSlater, ClarkeGuilliams, MartinSlavin, KonstantinGuinamard, RomainSlawson, ChadGuix, Francesc XavierSliwinska, AgnieszkaGujar, Amit D.Słomińska-Wojewódzka, MonikaGüldiken, NurdanSmadja, DavidGullberg, DonaldSmahel, MichalGundamaraju, RohitSmani, TarikGundersen, Cameron B.Smerage, Jeffrey B.Gunner, Marilyn R.Smetana, KarelGunter, Jennifer H.Śmieszek, AgnieszkaGuo, ChuanyongSmith Callahan, Laura A.Guo, SiqiSmith, Cody J.Guo, WeiSmith, Gaynor AnnGuo, YanSmith, Graham R.Guo, Zong ShengSmith, JessicaGupta, RajatSmith, Micholas DeanGupta, SanjaySmith, RobertGurevich, Vsevolod V.Soares, RaquelGurha, PriyatanshSobacchi, CristinaGuriec, NathalieSobierajska, KatarzynaGurskaya, Nadya G.Sobol, RobertGutierrez-Aguilar, ManuelSoibam, BenjaminGutiérrez-Juárez, RogerSokoloski, KevinGutierrez-Merino, CarlosSolana, RafaelGutsaeva, DianaSoler Valls, Ana JosefaGuyot, BorisSolovei, IrinaGyöngyösi, MariannSommariva, MicheleHa, PatrickSomoza, RodrigoHaack-Sørensen, MandanaSonawane, AbhijeetHaaparanta-Solin, MerjaSone, KenbunHaapasalo, AnnakaisaSong, ChunhuaHaas, Joel T.Song, JianxunHackl, HubertSong, SihongHaddad, Ahmed Q.Song, SuHadden, KyleSoni, DheerajHadjiargyrou, MichaelSoras, MichaelHadwiger, Jeffrey A.Sorci, GuglielmoHafizi, SassanSoreq, HermonaHafizi, SinaSorge, MatteoHäfner, NormanSorgi, Carlos ArtérioHagedorn, ClaudiaSoriani, AlessandraHahn, OliverSoriano-Arroquia, AnaHaining, Robert L.Sorrell, J. MichaelHakami, RaminSosna, JustynaHakkinen, LariSoto-Reyes, ErnestoHakovirta, Harri H.Soto-Reyes, IleanaHalász, MelindaSouček, PavelHalbrook, ChristopherSoudeyns, HugoHamacher, JürgSoudeyns, HugoHamamura, KazunoriSoundararajan, MeeraHamilton, Duane H.Sounni, Nor EddineHamilton, Matthew J.Soupart, AlainHampel, DanielaSoveral, GraçaHan, Jeung-WhanSpagnuolo, GianricoHanba, Yuko T.Spakova, TimeaHancock, MeaghanSpecht, AlexandreHanisch, Franz-GeorgSpendiff, SallyHannoun, ZaraSperti, CosimoHantusch, BrigitteSpinelli, Francesca RomanaHanukoglu, IsraelSpizzo, GilbertHao, JiaqingSpooner, RobertHao, MingangSpring, KevinHao, YajingSprio, Andrea ElioHaqshenas, GholamrezaSredni, Simone T.Hara, Emilio SatoshiSripathy, SmithaHarada, HisashiSrivastava, RiteshHarada, MasakoSrivastava, SaurabhHarari, DanielSt John, James A.Hardwick, James P.Stacchiotti, AlessandraHare, Joshua M.Stachewicz, UrszulaHargreaves, IainStagljar, IgorHarnett, WilliamStål, Per I.Haro, DiegoStanley, Jone A.Harper, JamesStanley, RobinHarrell, J. ChuckStark, Azadeh T.Harris, Andrew R.Staruschenko, AlexanderHartig, Sean MichaelStasiak, AndrzejHartman, MariuszStasyk, TarasHarwood, Adrian J.Stathopoulos, ConstantinosHashem, YaserStavolone, LiviaHashemolhosseini, SaidStavraka, CharaHashimi, HassanStecca, BarbaraHashimi, SaeedSteger, GerhardHashimoto, MakotoStegmaier, JohannesHashimoto, MasayukiStehbens, SamanthaHaspel, NuritStehle, JörgHassan, QuamarulStein, ColleenHassan, Sherif T. S.Steinberg, FlorianHata, YutakaSteinbichler, TeresaHattori, ToshioSteinbrenner, HolgerHaugsten, EllenSteinle, JenaHauser, PeterStelcer, EwelinaHautefeuille, MathieuStellato, FrancescoHayasaka, HarukoStenbeck, GudrunHayashi, MakotoStendahl, OlleHayashi, Mariko KatoStéphanie, Miserey-LenkeiHayashi, ShinichiroStepien, EwaHayashi, YoheiStevens, StanleyHazawa, MasaharuSteward, RobertHazrati, Lili-NazStewart, Colin L.He, ChunboStewart, JasonHe, FengStiburkova, BlankaHe, JingStilli, DonatellaHe, PeijianStinghen, AndréaHe, XiaochenStobiecka, MagdalenaHeales, SimonŠtorchová, HelenaHealey, GarethStornaiuolo, MarianoHeaphy, ChristopherStossi, FabioHebbard, LionelStott, Jennifer B.Hecker, MichaelStout, RandyHedtrich, SarahStrano, SabrinaHefferon, Kathleen L.Strappazzon, FlavieHegemann, PeterStratmann, BerndHeideman, WarrenStratton, DanHeinbockel, ThomasStroffekova, KatarinaHeise, Rebecca L.Stroh, AlbrechtHeitz, ThierryStrohbach, AnneHelbig, KarlaStuder, MIchèleHeldin, Carl-HenrikStukenborg, Jan-BerndHellerbrand, ClausStylianopoulos, TriantafyllosHellman, UrbanStylianou, AndreasHellmann, Jason L.Su, HuaHemmerich, PeterSu, MengHempel, UteSu, YeuHeng, Henry H.Subramanian, ArulHenkel, JaninSubramanian, ChitraHenriksson, JohanSud’ina, Galina F.Henry, Ryan A.Sudhoff, HolgerHer, Lu-ShiunSühs, Kurt-WolframHerbst, RuthSuk, KyounghoHermanns, HeikeSukocheva, OlgaHernandez, ArturoSukseree, SupawadeeHernández-Torres, FranciscoSukumar, PiruthiviHerraez, ElisaSulek, AnnaHerrero-Agustín, José-LuisSullivan, ConHerrin, DavidSullivan, Lucy C.Herrmann, MartinSumagin, RonenHershfinkel, MichalSun, ChenHervouet, EricSun, GongqinHess, AngelaSun, JianjunHetman, MichalSun, RamonHeumann, RolfSun, WeijingHewitt, AlexSun, YeHickson, Gilles RXSunami, YoshiakiHidalgo Pitaluga, LucasSundararajan, RajiHiebert, PaulSurguchov, AndreiHieda, MikiSurmenev, RomanHiggins, Paul J.Šušor, AndrejHiguchi, KeiichiSussman, Mark A.Hii, Charles S.Sutherland, BradHilhorst, MarcSutherland, James D.Himeno, HyoutaSuzuki, AussieHinds, Terry D.Suzuki, NorioHines, Dustin J.Svirshchevskaya, ElenaHippo, YoshitakaSvoboda, KathyHirasaka, KatsuyaSwann, KarlHirasawa, NoriyasuSweeney, TrevorHirsch, EmilioSwierczynski, JulianHirschi, KendalSydor, SvenjaHishimoto, AkitoyoSyková, EvaHlavaty, JurajSytykiewicz, HubertHochstrasser, TanjaSzanto, MagdolnaHodgkinson, ConradSzaryńska, MagdalenaHodoroaba, Vasile-DanSzatmári, TündeHoebeke, JohanSzeliga, MonikaHoehn, KyleSzeto, Yim-TongHoelzl, FranzSzöllősi, DánielHoffman, Robert M.Szoor, ArpadHoffmann, Markus H.Sztuba-Solinska, JoannaHoffmann, Peter R.Szulc-Dąbrowska, LidiaHofman, PaulTabolacci, ElisabettaHoggatt, JonathanTacke, FrankHohenester, Simon D.Tackenberg, ChristianHolefors, AnnaTaddei, Maria LetiziaHolien, TorilTafuri, AgostinoHollander, John M.Tagliabue, EldaHolly, JeffTaguchi, Y-h.Holnthoner, WolfgangTahirovic, SabinaHolsinger, DamainTaillandier, DanielHolstein, SarahTajiri, NaokiHolt, Scott M.Takada, IchiroHolzapfel, Gerhard A.Takagi, KiyoshiHolzmann, KlausTakahashi, HideyukiHomma, TakujiroTakahashi, ToshioHomma, TetsuyaTakaki, ManabuHong, Jiann-RueyTakakura, KazukiHong, TianpeiTakami, AkiyoshiHong, Yi-RenTakano, AtsukoHong, YonggeunTakano, JunpeiHoogewijs, DavidTakasawa, ShinHooper, CorneliaTakashi, SekiyaHori, OsamuTakashima, EizoHori, ToshiyukiTakayama, TadatoshiHorin, PetrTakeda, SenHorne, MaryTakegahara, NorikoHorowitz, John D.Takemori, HiroshiHorsburgh, AnnTakeuchi, KazuhikoHorváth, Eszter MáriaTalati, MeghaHoseini-Yazdi, HoseinTallquist, MichelleHoshi, AkihikoTalmadge, JamesHosick, PeterTalora, ClaudioHoskins, ClareTamaki, TetsuroHossain, Md KamalTambe, MitaliHou, PanpanTamkovich, SvetlanaHoushdaran, SaharTamura, RuiHouslay, MilesTan, KarenHoy, AndrewTan, Meng HowHribal, MartaTan, Tse-HuaHsiao, Hui-HuaTan, WenbinHsiao, Kuei-YangTan, YutingHsieh, Hsi-LungTanabe, ShihoriHsieh, Tusty-JuanTanaka, MinoruHsu, Tuan-TiTanaka, NaoroHsu, Yi-ChiungTanaka, SatoshiHu, Dong GuiTanaka, TakujiHu, GuokuTanase, CristianaHu, HongzhenTanca, AlessandroHu, JunhuiTandon, RiteshHu, Ke-qinTang, BorHu, PingzhaoTang, Chih-HsinHuang, Hsiao-YunTang, Dale D.Huang, Hsu-ShanTang, JingjingHuang, HuTang, KaiHuang, Hui-ChunTang, LongtengHuang, Jian-dongTang, QiyiHuang, Kuo-HowTang, YaohuiHuang, Sarah XuelianTaniguchi, KoheiHuang, StanleyTanimizu, NaokiHuang, Tzu-TingTanti, Goutam KumarHuang, VivianTantos, ÁgnesHuang, XiaohuTao, JunyanHuang, Ying-HsienTao, Ya-XiongHuang, YunlongTaoufik, ErasmiaHuber, RTarantino, GiovanniHuber, RobertTarasova, Nadya I.Hübner, ChristianTatakis, DimitrisHuda, NazmulTate, Rothwelle J.Hudcovic, TomasTatebe, HisashiHudson, Laurie G.Tatsuke, TsuneyukiHughes, SamanthaTatsumi, KoukoHugues, StéphanieTattikota, Sudhir GopalHuhn, RagnarTatullo, MarcoHui, WangTaube, JosephHuleihel, MahmoudTaura, KojiroHumphries, BrockTavassoly, ImanHung, Shao-WenTaylor, Alison MarieHunt, DavidTaylor, CarolineHuntosova, VeronikaTaylor, CormacHura, TomaszTchetina, Elena V.Husnjak, KoraljkaTe Velthuis, AartjanHussain, SamanTee, Andrew R.Huwiler, AndreaTelerman, AdamHwang, Gi-WookTellez-Gabriel, MartaHwang, In KooTencerova, MichaelaHyde, Ricia KatherineTeng, Ru-JengHyun, Sang-HwanTeng, ShaoleiIaccarino, IngramTeng, YongIaccino, EnricoTenner, Andrea J.Iacobazzi, VitoTeodoro, Jose G.Iadevaia, ValentinaTeotia, PoojaIdogawa, MasashiTerracciano, DanielaIdris, AdiTerrazas, Luis I.Ientile, RiccardoTerrinoni, AlessandroIeraci, AlessandroTerry, StéphaneIino, MasamitsuTerry, Stephen J.Ikeda, MasahiroTeruel, Mary N.Illes, ZsoltTerzaghi, William BryanIlmer, MatthiasTeschke, RolfIm, Seung-SoonTey, Siew LingImai, YoichiThackray, AlanaImamoto, YasushiThaker, Youg RajImamura, MasahiroThakurela, SudhirIncitti, TaniaThanigaimani, ShivshankarIneichen, BenjaminThenet, SophieInfante, ArantzaTheodoropoulos, GeorgeInfante, PaolaThomas, ChristoforosIorio, EgidioThomas, T.J.Iram, SurtajThompson, BarryIrigoyen, NereaThomson, David M.Irimura, TatsuroThongprayoon, CharatIrles, ClaudineThorne, PeterIrwin, David C.Thumm, MichaelIsaguliants, MariaTikellis, ChristosIshibashi, NobuyukiTilton, Susan C.Ishigaki, YasuhitoTimm, JörgIshihara, KatsuhikoTimmons, LisaIshikawa, TomohiroTirino, VirginiaIshikawa, YasuyukiTiriveedhi, VenkataswarupIshimoto, TakahiroTiso, NatasciaIsidoro, CiroTissenbaum, Heidi AIslam, Mohammad MirazulTiwari, Rakesh K.Isola, GaetanoTliba, OmarIsozaki, TakeoTobiasch, EddaItatani, YoshiroToca-Herrera, JoseItoh, SusumuToda, TakashiItoh, YoshifumiTodi, Sokol V.Iuliano, RodolfoTodryk, StephenIwai, AtsushiTodt, DanielIwamoto, NaokiToedebusch, ChristineIwamoto, ShotaroTogo, FumiharuIwasaki, YukaTogo, ShinsakuIwata, JunichiToh, Tan BoonIwata, KazumiToivanen, RoxanneIwata, TakanoriTokmakov, Alexander A.Izawa, TakeshiTolosa, EzequielIzawa, TetsuyaTolwinski, NicholasIzdebska, MagdalenaToma, ClaudioIzumi, NamikiTomar, DhanendraJ, Gil-MohapelTomàs, JosepJaaro-Peled, HannaTomassetti, AntonellaJablonska, EwaTomicic, MajaJablonska, KarolinaTomilin, Alexey N.Jacinto, EstelaTomita, YusukeJacobs, Frank M. J.Tomiyama, ArataJacomin, Anne-ClaireTomizawa, ShinichiJacquin, EliseTomuleasa, CiprianJaeschke, HartmutTong, XinJaguva Vasudevan, Ananda AyyappanTong, Yat-ChingJähn, KatharinaTönges, LarsJaimovich, EnriqueToniolo, LuanaJain, ShrutiToomer, Gabriela Chavez-CalvilloJaing, Tang HerTorras, JuanJaiswal, Ashvin R.Torre, Maria LuisaJaiswal, DeepikaTorres, MagdalenaJaiswal, J.K.Torres-Lagares, DanielJakubowski, HieronimToruner, Gokce AltayJalkanen, SirpaTósaki, ÁrpádJames, DuceTosato, GiovannaJameson, PaulaToshimitsu, YamaokaJamieson, Stephen M. F.Tosi, SabrinaJanczar, SzymonTóth-Molnár, EditJandl, KatharinaTothova, CsillaJaneczko, MonikaTotta, PierangelaJang, Sung KeyTourniaire, FranckJanjetovic, ZoricaTowers, ChristinaJantas, DanutaToya, YoshihiroJanuszkiewicz-Lewandowska, DanutaToyoda, MasashiJarome, TimothyToyoda, YuJasiecki, JacekToyoma, TadashiJaskula-Sztul, RenataTozzi, Maria GraziaJaumot, JoaquimTrajkovic, VladimirJauregui, CatherineTrammell, SamJavadov, SabzaliTran, Anh NhatJay, StevenTrant, JohnJayant, RahulTravelli, CristinaJayaprakash, PriyamvadaTrebicka, JonelJayaraj, RamaTremblay, JacquesJean, DidierTremblay, Jacques P.Jekabsone, AisteTremblay, KimberlyJembrek, Maja JazvinšćakTrichet, ValérieJeney, ViktoriaTrinchera, MarcoJeng, Jiiang-HueiTripathi, Jitendra KumarJeon, Sang-MinTrivedi, Meghana VJeong, Keun-YeongTrivedi, RachanaJerebtsova, MarinaTroiano, GiuseppeJeronimo, CarmenTroidl, KerstinJeschke, UdoTrubiani, OrianaJetten, AntonTruong, Vi KhanhJeung, Eui-BaeTsai, Ang-ChenJewell, JennaTsai, KelvinJeyaseelan, SamithambyTsai, RongkungJia, DongyaTsakiridis, AnestisJia, YulinTsang, WilliamJiang, Hui-RongTsapogas, PanagiotisJiang, Lin-HuaTsirigos, KonstantinosJiang, Qiu-XingTsourdi, ElenaJiang, YanyanTsoyi, KonstantinJimenez, WladimiroTsuchiya, AkiraJiménez-Mateos, Eva MaríaTsuda, TakeshiJimi, EijiroTsukahara, ToshifumiJin, KunlinTsukamoto, TetsuoJin, XingzhongTsukiyama-Kohara, KyokoJing, RanTu, ThomasJochum, ChristophTu, ZhidongJohansen, KristenTucker, LarryJohansson, PegahTuettenberg, AndreaJohnson, DanielTulis, David A.Johnson, GregoryTuller, TamirJohnson, JohnTulotta, ClaudiaJohnstone, DanielTuma, PamelaJolly, M.K.Tunaru, SorinJonsson, Anna HelenaTuomela, JohannaJoo, Kyeung MinTurco, JeniferJorgačevski, JernejTuretta, MatteoJosef, KapfhammerTurley, Eva A.Joshi, AmitTurner, Alice M.Jost, Wolfgang H.Turner, DanJoyce, Michael GordonTurner, Jason D.Juarranz, YasminaTurner, NeillJucaite, AurelijaTuttolomondo, AntoninoJugo, Begoña M.Tuura, Ruth O’GormanJuhász, GaborTuyaerts, SandraJuhász, TamásTyagi, RahulJun, Chang-DukTyagi, SonikaJung, Alain C.Tyan, Yu-ChangJung, JangwookTylzanowski, PrzemkoJung, Ji HoonTyrrell, LorneJung, KlausTzur, AmitJung, Sung ChulUchio, YujiJungnickel, HaraldUchiumi, ToshioJura, JolantaUdono, HeiichiroJurjus, Rosalyn A.Ueno, NaotoJurk, KerstinUeno, Shu-ichiJurkowska, HalinaUgarte-Berzal, EstefaniaJurkowska, Renata ZofiaUldrijan, StjepanJurman, GiuseppeUlivieri, CristinaJyotsna, MishraUlrich, MagdaKabakov, Alexander E.Umemura, NaokiKabashi, EdorUngefroren, HendrikKaddour, HusseinUnger, MarcusKaessmeyer, SabineUngerstedt, Johanna S.Kaether, ChristophUngurianu, AncaKaigorodova, EvgeniyaUnni, Arun M.Kaikkonen, Minna UUray, IvanKailu, YangUrbanelli, LorenaKaitsuka, TakuUriarte, IkerKaji, KosukeUribarri, JaimeKajiya, HiroshiUrra, FelixKajiya, MikihitoUshimaru, TakashiKakkola, LauraUsmani, ShariqKalani, AnuradhaUygun, SahraKalani, KomalUzbekov, Rustem E.Kalasekar, SharanyaUziel, OritKale, AbhijitV Ivanov, AlexanderKalló, GergőV. González López, CynthiaKallunki, TuulaVaandrager, Arie B.Kalogirou, CharisVacher, PierreKaltschmidt, BarbaraVachtenheim, JiriKamada, YoshiakiVaidyanathan, VenkateshKamath, DivyaVaira, ValentinaKamato, DanielleValdes-Lopez, OswaldoKamensek, UrskaValente, CarmenKamihira, MasamichiValenti, MariaKaminski, RafalValentine, Rudy J.Kammerer, RobertValius, MindaugasKanamori, TakaoVallée, AlexandreKanazawa, MasatoValtink, MonikaKanbayashi, TakashiValverde, A´ MKanda, TatsuoVan Breda, EricKanekiyo, TakahisaVan Bussel, Bas C. T.Kaneko, KaichiVan De Stolpe, AnjaKang, DongchulVan den Noort, MauritsKang, HunseungVan Der Vorst, EmielKang, JunsuVan Esch, Betty C. A. M.Kang, Min-JungVan Gent, DikKang, Sang SunVan Horn, WadeKang, Tae-BongVan Kasteren, PuckKang, Tong HoVan Loon, BarbaraKang, Un Jungvan Vugt, MarcelKang, WeiVandenbroeck, KoenKang, Young-HeeVandenbroucke, Roosmarijn E.Kang, Yunqing (Kevin)Vanderleyden, IneKango-Singh, MadhuriVanhaesebroeck, BartKanisicak, OnurVaquero, AlejandroKano, YutakaVarchetta, StefaniaKant, SashiVarga, GeorgKanugula, AnanthaVarga, MátéKao, Shu-HueiVarga-Weisz, PatrickKapetanaki, MariaVarghese Gupta, SheebaKapogiannis, DimitriosVárkonyi, JuditKapoor, MohitVaschetto, RosannaKar, AdwitiyaVasconsuelo, AndreaKarabina, Sonia-AthinaVasicek, OndrejKaragiannis, SophiaVassalle, CristinaKarantanos, TheodorosVassallo, NevilleKarasiński, JanuszVassiliou, Aliki GeorgiaKardassis, DimitrisVaughan, RogerKariya, YoshinobuVaughn, DavidKarlsson, ErikVázquez, PatriciaKaroor, VijayaVecellio, MatteoKarwowski, Boleslaw T.Vechetti, IvanKasahara, AtsukoVecoli, CeciliaKasai, FumioVeenman, LeoKasparov, SergeyVeeramani, SureshKass, LauraVega, Francisco M.Kasten, Benjamin B.Végh, Attila GergelyKatada, KazuhiroVeis, Deborah J.Kataoka, NaoyukiVeit, GuidoKato, NaohiroVelasco-Hernández, TalíaKato, ShigeakiVeldkamp, ChristopherKato, Takamitsu AVenkatesan, ThiagarajanKatsavelis, DimitriosVenkateshaiah, Sathisha U.Katyal, PriyaVerde, GaetanoKauppinen, AnuVergani, PaolaKawakami, SusumuVergara, DanieleKawakami, ToshiVerghese, ShilpiKawauchi, TakeshiVerma, Dinesh KumarKazanietz, MarceloVerma, PriyankaKazeto, YukinoriVerma, SureshKe, Liang-YinVerma, Suresh KumarKe, Po-YuanVermeer, Paola D.Ke, YouqiangVermeulen, LouisKeane, Robert WVerrecchia, FranckKebriaei, PVersacci, PaoloKeenan, JoanneVicente, CristinaKello, MartinVictor, VictorKelly, Alexander E.Vidal, ReneKemp, Michael G.Vidavsky, NettaKemp, RoslynViel, SébastienKempisty, BartoszViennois, EmilieKendall, TimVieyra, AdalbertoKenneth, NiallViganò, MarcoKenny, ParaicVigneri, PaoloKerr, IanVignes, MatthieuKerwin, Rachel E.Vijayan, MadhuvanthiKetkar, AmitVila, MiquelKhajavi, NoushafarinVilain, SébastienKhalyfa, AbdelnabyVilla, ChiaraKhambu, BilonVillalobos, CarlosKhammanivong, AliVillarreal Molina, Maris TeresaKhedoe, Padmini S.J.Villegas, VictoriaKhodanovich, MarinaVilloslada, PabloKhoo, Bee LuanViñals, FrancescKhozin-Goldberg, InnaVincentini, OlimpiaKhuchua, ZazaVinciguerra, ManlioKi, Sung HwanVinnakota, Kalyan C.Kiecker, ClemensVirag, LaszloKieda, ClaudineViranaicken, WildrissKietzmann, ThomasVisser, LydiaKilari, SreenivasuluVitetta, LuisKim, BongleeVivacqua, AdeleKim, Byung SooVivo, MariaKim, Cheorl-HoVizi, E. SylvesterKim, DaeVizoso, Francisco J.Kim, Deok-HoVladimirov, VladimirKim, Dong-HweeVlajnic, TatjanaKim, HangunVliagoftis, HarissiosKim, Hee-SikVoena, ClaudiaKim, Hyung SikVogel, MoniqueKim, Jae YoungVogt, Leonie MarloesKim, JeongkwonVolarevic, VladislavKim, Jin-WookVolpe, ElisabettaKim, JongminVolta, MattiaKim, JunghyunVon Buchwald, ChristianKim, KyounghyunVon Eyss, BjoernKim, LouVon Laer, DorotheeKim, MyungjinVon Schwarzenberg, KarinKim, Sang SooVoss, MatthiasKim, Sang-WeVugrek, OliverKim, Seong-GonVychytil, AndreasKim, Seong-KyunWachten, DagmarKim, Sun JungWada, Ken-IchiKIM, Sung JoonWagner, GunterKim, Tae-HyungWagner, Kay DietrichKim, Tae-JungWahli, WalterKim, YanghaWahli, WalterKim, Yong-GilWairkar, Yogesh P.Kim, YoungsooWalch, PatrickKimura, KiminoriWalczak, ClaireKimura, TsuyoshiWalker, Chandler L.Kind, JopWalker, ValerieKinugasa, HideakiWall, DanielKioussi, ChrissaWall, NathanKipp, MarkusWallace, Nicholas A.Kirchmaier, AnnWalldorf, UweKirischuk, SergeiWalther, WolfgangKirschnek, SusanneWalz, JulianeKirschner, KristinaWan, EdwinKiss, Anna L.Wan, JunKiss, AttilaWan, Lam YimKiss, RobertWan, ShibiaoKitagawa, SeiichiWandosell, FranciscoKitaura, JiroWang, Bang-AnKitchen, PhilipWang, Chih-KuangKittel, AgnesWang, ChihueiKjaerulff, OleWang, Chrong-ReenKlaassen, IngeborgWang, ChunKlar, AgnesWang, GuliangKlaus, SusanneWang, GuozhengKlein, UlfWang, HaichaoKleiter, NataschaWang, HsiuyingKlemm, RobinWang, JianmingKlimaszewski, LarsWang, JingKlimczak, AleksandraWang, Ju-MingKlionsky, DanielWang, KaiKloc, MalgorzataWang, KankanKloskowski, TomaszWang, QingjunKlupp, BarbaraWang, RanranKnäuper, VeraWang, RuoxiangKnezevic, JelenaWang, ShaobinKnittelfelder, OskarWang, WenqiKnoll, MarkoWang, XiaodongKnoop, VolkerWang, XinwenKo, Jane L.Wang, Yi-GangKobayashi, Eri H.Wang, Yi-zhiKobayashi, SatoruWang, YongqiangKobayashi, TatsuyaWang, YoujinKobeissy, FirasWang, YumengKobold, SebastianWang, YumengKočí, ZuzanaWang, ZhenboKodet, OndrejWang, ZhenpingKodidela, SunithaWang, ZhixiangKoga, FumitakaWanrooij, SjoerdKoga, YoshikatsuWard, Marie-LouiseKohaar, InduWard, Peter A.Kohanbash, GaryWardill, Hannah R.Köhl, GudrunWasilewska, BarbaraKohno, NobuakiWatanabe, KazuhideKoike, ShinWatanabe, ToshioKoivunen, PeppiWaters, PaddyKoizume, ShiroWatt, SuzanneKojima, MasamiWeaver, JolantaKokkaliaris, KonstantinosWebb, R. ClintonKolesnikova, TatyanaWeber, FriedemannKolettas, EvangelosWeber, Georg F.Kolonin, Mikhail G.Weber, ViktoriaKolosov, PeterWebster, Nicholas J.G.Komatsu, MasanobuWegwitz, FlorianKomis, GeorgeWei, Hong-JianKomiyama, TomoyoshiWei, ShouKommireddy, VasuWeigand, Julia E.Kondo, JumpeiWein, Marc N.Kondo, TatsuyaWeinberger, FlorianKong, Il-KeunWeinhold, NielsKong, WeiliWeintraub, NealKonstantinidis, Theocharis G.Weiser, Daniel A.Kontoravdi, CleoWeiss, Mark L.Kónya, JózsefWeiss, Thomas S.Kopel, PavelWelsh, JaneKops, GeertWelsh, MichaelKorac, BatoWelshhans, KristyKoralov, SergueiWelters, Hannah J.Korfei, MartinaWen, JianjunKörner, CindyWen, ZhiweiKorona, DagmaraWenbin, ZhanKorsching, EberhardWenkel, StephanKortholt, ArjanWerley, Christopher A.Kosinsky, Robyn LauraWerner, HaimKotsikorou, EvangeliaWesche, JørgenKottilil, ShyamWestenfelder, ChristofKotula-Balak, MałgorzataWesthauser, FabianKoturbash, IgorWestmark, CaraKouza, MaksimWetzel, ChristianKovács-Öller, TamásWhite, FletcherKowalczyk, Anne E.Wibowo, DavidKowtharapu, Bhavani S.Wicky Collaud, ChantalKozar-Kaminska, KatarzynaWieczorek, EdytaKoziak, KatarzynaWiese, StefanKozma-Bognár, LászlóWigle, JeffreyKracht, MichaelWilce, JackieKrahmer, NatalieWilley, ChristopherKratochvíl, LukášWilliams, BrianKraus, ArminWilliams, Jessica A.Kraus, PetraWilliams, JoannaKrejbich-Trotot, PascaleWilliams, RichardKremling, AndreasWillis, RohanKretz-Remy, CaroleWilson, Derek J.Krick, StefanieWiltshire, Esko J.Kriegel, AlisonWin, Khin YinKrijt, JanWinship, AmyKrisher, Rebecca LWinter, StephanKrishnakumar, ShyamWischhusen, JörgKrishnamurthy, AkilanWitkowska, Anna MariaKristian, TiborWittmann, JürgenKriz, JanWittner, LuciaKrohne, GeorgWłoga, DorotaKrupa-Kozak, UrszulaWobus, ManjaKrupenko, Natalia I.Wodrich, HaraldKruspig, BjornWoeller, CollynnKrysov, SergeyWojtunik-Kulesza, Karolina A.Krzymowska, MagdalenaWöllert, TorstenKu, NamonWong, Chi-HueyKuang, ShihuanWong, Chi-MingKubaczka, CarolineWong, Chi-MingKubatka, PeterWong, Ching OnKubinová, ŠárkaWong, Hovy Ho-WaiKuboki, TakuoWong, RaymondKucheryavykh, Lilia Y.Wong, Tzu-TsungKuchler, KarlWoo, So-YounKuçi, SelimWoolford, JohnKudo, YasuseiWosczyna, MichaelKudoyarova, GuzelWree, AlexanderKudryashov, Dmitri S.Wu, ChengbiaoKuehl, MichaelWu, ErxiKues, Wilfried A.Wu, JiandongKugler, Jan-MichaelWu, Li-chenKühl, Anja A.Wu, MingfuKukley, MariaWu, Re-WenKulakovskaya, Tatiana V.Wu, Tzong-YuanKulasinghe, AruthaWu, XianfangKulik, AnnaWu, XinKulp, SamuelWu, YadiKulski, Jerzy K.Wu, Yih-RuKumar, A. PratapWu, Yu-JenKumar, AkhileshWulff, HeikeKumar, BinodWullaert, AndyKumar, GauravWurdak, HeikoKumar, RameshWürth, RobertoKumar, SanjeevXian, WaKumar, SunilXiao, LiKumar, UjendraXiao, XiangweiKumar, VijayXie, WankunKumari, SudhaXing, JianhuaKunej, TanjaXing, JunjiKunz, WolframXirodimas, DmitrisKUO, Cheng-ChinXu, DazhongKurtenbach, StefanXu, HongKuryłowicz, AlinaXu, HongyanKusaczuk, MagdalenaXu, JianKusamori, KosukeXu, JingKusner, Linda L.Xu, KuiKuśnierczyk, PiotrXu, MeifengKuwano, KazuyoshiXu, QinZiKveiborg, MarieXu, XinKwak, Hyo-BumXu, YanKwak, Jong HwanXu, YuexinKwakowsky, AndreaXu, YungangKyriazis, George A.Xu, ZhenLa Mendola, DiegoXue, BingzhongLa Mura, VincenzoYadav, MarshleenLa Rosa, MassimoYagci, TamerLacham-Kaplan, OrlyYager, EricLacina, LukasYagi, HiroshiLadanyi, AndreaYagihashi, SorokuLadewig, JuliaYago, ToruLadilov, YuryYamada, HiroyukiLadzynski, PiotrYamada, TaketoLafontaine, DenisYamamoto, YusukeLaforenza, UmbertoYamamura, SoichiroLagendijk, Anne KarineYamanaka, RyuyaLagger, SabineYamano, KojiLaghezza, AntonioYamasaki, MasahiroLahiri, AmitabhaYamashita, AtsushiLahlil, RachidYamatsu, KojiLai, Jui-YangYa-Ming, ChengLai, Michael M. C.Yammani, Raghunatha R.Lakshmanan, ImayavarambanYan, DongqingLal, HindYan, ShengminLalli, EnzoYan, XuLallier, Thomas E.Yanase, SuminoLalowski, MaciejYang, Chih-JenLam, RaymondYang, Deng-HoLam, YanYang, GuangLam, Yuen TingYang, HongyuanLamarche-Vane, NathalieYang, JialeLamas, J. AntonioYang, JinzengLamas, José RamónYang, Jun-YiLamb, JacobYang, KunLamort, Anne-SophieYang, LiuLampson, Michael A.Yang, PengyuanLancel, SteveYang, Shang TiangLanfrancone, LuisaYang, TaoLang, DiYang, WancaiLang, Jennifer K.Yang, XiaoheLang, RolandYang, Ying-YingLang, StephanYannarelli, GustavoLangdon, SimonYano, HajimeLange, TobiasYao, JiayiLangner, CordYao, MinLanning, NathanYao, ZhenqiangLaoui, DamyaYarowsky, PaulLaPres, John J.Yasoda, AkihiroLara, LucienneYasuda, JunLarauche, MurielYatoh, ShigeruLarijani, ManiYazawa, TakashiLarre, IsabelYe, KeqiangLarschan, EricaYe, WenleiLasagni, LauraYeh, Chi-TaiLascano, JorgeYeh, I-JuLassmann, Hans P.Yeh, Jwu-LaiLattanzi, GiovannaYeo, GiselleLau, Chung-Ho EsmondYeste, MarcLaudisi, FedericaYeung, Tsz-LunLauersen, KyleYew, P. ReneeLauf, PeterYi, Sang AhLaurent, GarderetYi, Young-SuLaurent, MichaëlYilmaz, ÖzlemLauretani, FulvioYin, ChunyueLauronen, JouniYip, Bon HamLauschke, Volker M.Yip, Calvin K.Lauth, MatthiasYlostalo, Joni H.Lavogina, DarjaYochum, Gregory S.Lavoie-Cardinal, FlavieYoder, Jeffrey ALavrentieva, AntoninaYokota, TakafumiŁawicki, Sławomir ł.Yokoyama, Kazunari K.Lawson, CharlotteYoneda, ToshiyukiLawson, VictoriaYonekura, HidetoLax, PedroYoneshiro, TakeshiLaxa, MiriamYoo, ChanghoonLayer, Paul G.Yoo, Yeong-MinLazarević, VladimirYool, AndreaLazo-Zbikowski, PedroYoon, IlLe Blanc, KatarinaYoshida, KatsunoriLe Goff, AnneYoshie, MikihiroLe Stunff, HerveYoshifuji, HajimeLe, WeidongYoshikawa, KeisukeLeabu, MirceaYoshimura, MasamiLebaron, RichardYoshino, HironoriLebaron, SimonYoshioka, Ken-ichiLebrasseur, OphélieYoshioka, WataruLechel, AndreYou, Hye JinLederkremer, Gerardo Z.You, HyunjuLee, Chang HoonYou, SeungkwonLee, ChangWooYoung, Howard A.Lee, Chia-HwaYousefi, ShidaLee, Dae HoYu, Han-GangLee, Dong SoonYu, LuLee, Dung-FangYu, SiwangLee, ErinnaYu, VictorLee, Huei-JaneYu, WenboLee, Jae-HoYu, YuLee, JeeyoungYuan, XuegangLee, Jun SikYuan, ZhihongLee, JungwooYue, JunmingLee, Ju-SeogYui, NaofumiLee, KevinYun, ChrisLee, Kyung-AhYun, Jong WonLee, Moo-SeungYun, MiyongLee, Sheng-AnYung, Hong-WaLee, Tse-MinYuseff, Maria-IsabelLee, Tsung-HsienYushenova, IrinaLee, Tzong-ShyuanYuste, Victor J.Lee, Yeon SunYves, CombarnousLee, Yu-HsuanZabetakis, IoannisLegembre, PatrickZaccai, MicheleLegouis, RenaudZacchigna, SerenaLehmann, MaximeZachow, StefanLeitch, HarryŻaczek, Anna J.Leite, KatiaZádor, ErnőLeitinger, BirgitZádori, ZoltánLeng, RogerZagórska, AgnieszkaLens, SabelaZahedi, KamyarLenz, Tobias L.Zahid, MalihaLenzi, PaolaZaid, HilalLeón, JosefaZaidi, SobiaLeonetti, CarloZaiss, DietmarLeporatti, StefanoZallot, Remi G.Leprince, CorinneZaman, AubhishekLesniak, WieslawaZamay, Anna S.Letizia, TrovatoZampese, EnricoLeucci, EleonoraZander, MarkLeung, RickyZang, LiqingLevantini, ElenaZannetti, AntonellaLevin, David E.Zanone, Maria M.Leviyang, SivanZanussi, StefaniaLevy, Arkene S.A.Zapico, Sara C.Levy, Finn OlavZapico, Sara CasadoLevy, MichaelZappulla, DavidLewellyn, LindsayZaravinos, ApostolosLewin, AlfredZariwala, Maimoona A.Lewis, MartinZarogoulidis, PaulLezoualc’h, FrankZavan, BarbaraLezza, Angela Maria SerenaZdzieblik, DeniseLi, ChaoZegers, Mirjam M.P.Li, Chia-JungZeilinger, CarstenLi, ChuanfuZelensky, Alex N.Li, FengZelles, TiborLi, Hsing-HuiZeltner, NadjaLi, HuaZenarruzabeitia, OlatzLi, HuinanZeng, GuangmingLi, JianrongZeng, HuLi, Jiao JiaoZenker, SvenLi, JunZhai, KuiLi, LiZhan, XuanzhiLi, LianboZhan, YiqiangLi, LingZhang, BaolinLi, LingxiaoZhang, DianbaoLi, MingqiangZhang, DuoLi, QiaoliZhang, HuijieLi, Ronald H.L.Zhang, JianminLi, Shengwen CalvinZhang, JianyingLi, ShitaoZhang, JingyuLi, SunanZhang, JinweiLi, TianhuZhang, JunranLi, WeiZhang, LiLi, WeihanZhang, QianLi, WenboZhang, QingLi, XiangZhang, ShulingLi, XiaohongZhang, SicongLi, XiaotaoZhang, WeijieLi, YanZhang, XuexinLi, YanZhang, YijunLi, YangZhang, Yin HuaLi, ZheZhang, YixiangLian, QizhouZhang, YixuanLiao, Jiahn-HaurZhang, YouyiLiao, Jyh-feiZhang, YuminLiao, PengZhao, JianfeiLiao, Yi-JenZhao, Jie JaneLi-Beisson, YonghuaZhao, LinlinLibra, MassimoZhao, LiqinLiby, KarenZhao, QingnanLichocka, MałgorzataZhao, XiaoaiLichtenauer, MichaelZhao, YunfengLidgerwood, GraceZhao, ZhiminLiebau, StefanZheng, GuopingLiebl, FaithZhong, CuiqingLieder, BarbaraZhong, ZhaohuaLiesveld, JaneZhou, BeiyanLim, Kee SiangZhou, BeiyunLim, Kyung-MinZhou, ChangchengLima, Clélia Akiko HirumaZhou, HaiyanLimonta, DanielZhou, HuitongLin, Chih-LiZhou, JichunLin, Chi-HungZhou, JingyingLin, Dar-ShongZhou, XiaoboLin, HoZhou, XiaodongLin, Huey-JenZhou, ZhanxiangLin, Kuan HungZhou, ZhidongLin, Kwang-HueiZhu, Michael X.Lin, RongtuanZhu, XueweiLin, ShellyZhukova, NataliaLin, WeichunZi, XiaolinLin, WenshengŽiarovská, JanaLin, WenyuZielenkiewicz, PiotrLin, Wey-jinqZienkiewicz, KrzysztofLindahl, LasseZingg, Jean-MarcLindner, RobertZiolkowski, WieslawLindsay, Andrew J.Zolnerowich, GregoryLindsey-Boltz, LauraZorov, Dmitry B.Lindstrom, Jon MartinZou, QuanLindström, Mikael S.Zsarnovszky, AttilaLinert, WolfgangZubiaga, AnaLink, BrianZugaza, J. L.Liobikas, JuliusZunke, FriederikeLionetti, LillàZuppinger, ChristianLiou, Chian-JiunZuryn, StevenLippert, ElisabethZwacka, RalfLipstein, Noa


